# Deactivation and Regeneration of Lewis Basic Sites Following Reversible Chemical Adsorption and Desorption of Hydroxyl Groups in Contaminant Degradation by Advanced Oxidation

**DOI:** 10.3390/ma19081589

**Published:** 2026-04-15

**Authors:** Lekang Zhao, Huailin Fan, Juncheng Zhao, Xixi Zhang, Xiaohang Ma, Xun Hu, Qingyu Ma

**Affiliations:** School of Material Science and Engineering, University of Jinan, Jinan 250022, China; lekangzhao001@163.com (L.Z.);

**Keywords:** reutilization of spent catalysts, advanced oxidation, reversible chemical adsorption and desorption, Lewis base, deactivation and regeneration

## Abstract

The Lewis basic catalysts were susceptible to poisoning during the activation of peroxymonosulfate, resulting in their transformation into spent catalysts and subsequent secondary environmental contamination. In this work, the chemical constitution of the catalyst’s surface during both the deactivation and regeneration processes was intensively tracked. The mechanistic studies revealed that the reversible bonding of adsorbed hydroxyl groups generated from peroxymonosulfate activation with Lewis basic carbon atoms adjacent to pyridinic nitrogen was identified as the intrinsic mechanism responsible for the catalyst regeneration, accompanied by the reappearance of Lewis basic sites. Following high-temperature or sodium borohydride reduction, the activity of the catalysts was restored to over 90% of the initial activity, enabling the spent catalysts to be reused multiple times. Catalyst deactivation corresponded to an increase in the C–OH content from 24.3% to 35.2%, whereas regeneration reduced it to 25.16%. Furthermore, a strong inverse correlation was observed between the surface hydroxyl density and the catalytic activity. The study elucidates the deactivation and regeneration mechanisms of Lewis basic catalysts at the atomic scale, paving the way for durable applications in advanced oxidation processes.

## 1. Introduction

With the rapid development of industrial production and human activities, the content of organic pollutants detected in water supply systems has increased year by year [1,2]. These pollutants include dyes, antibiotics, endocrine disruptors, and pesticides. These pollutants not only inhibit the photosynthesis of aquatic plants but also contain numerous carcinogens and their decomposition products, which could cause carcinogenic and teratogenic effects on public health, posing a significant risk [3]. The primary approaches employed for the removal of organic contaminants from wastewater include adsorbent adsorption, reverse osmosis filtration, advanced oxidation processes (AOPs), and electrochemical oxidation. AOPs generate various highly oxidizing active species, enabling rapid degradation of organic contaminants into non-toxic or low-toxic substances [4]. This is considered one of the most effective approaches for treating wastewater. Persulfate-based advanced oxidation processes (PS-AOPs) have emerged as the preferred strategy for remediating recalcitrant organic pollutants, thanks to their high oxidation potential (2.5–3.1 V), extended half-life (30–40 µs), and ability to adapt to complex water matrices [5].

Despite its excellent performance, peroxymonosulfate (PMS) exhibits poor self-decomposition capability due to its high decomposition activation energy (the O-O bond energy being approximately 140–213 kJ/mol) [6]. This characteristic results in insufficient activation kinetics during practical applications [7]. Consequently, the utilization of catalysts is necessary to reduce the decomposition activation energy of PMS, thereby accelerating the activation process. Wang et al. demonstrated that catalysts could effectively lower the energy barrier for O-O bond cleavage within PMS, facilitating its decomposition [8]. Carbon-based catalysts became highly promising catalytic materials due to their wide availability, low cost, and environmental friendliness. Among these, biomass, including rice straw, spirulina, animal excrement, sewage sludge, and cotton, was selected as a valuable precursor for synthesizing biochar-based catalysts due to its abundance and sustainability. Its abundant functional groups and well-developed porous structure conferred significant advantages for the synthesis of such catalysts. However, the inherent catalytic efficiency of carbon-based materials is relatively low. By leveraging the differences in atomic radius, orbital configuration, electron density, and electronegativity between heteroatoms (N, P, S, and B) and carbon atoms, the doping of heteroatoms into the carbon skeleton was implemented [9]. This process effectively modulated the electron density on the carbon surface, leading to the generation of new catalytically active sites and significantly enhancing the catalytic efficiency [10]. Among these approaches, nitrogen doping is recognized as the most effective method for enhancing catalytic activity because the nitrogen atoms are able to increase the electron spin density of adjacent carbon atoms [11]. This enhancement strengthens electrostatic adsorption between the catalyst and PMS, improving catalytic efficiency [12].

Among nitrogen-doped species, pyridinic nitrogen featuring Lewis basic sites is recognized as the catalytically active site [13]. The doping of pyridinic nitrogen modifies the local density of states of adjacent carbon atoms, resulting in the electrostatic adsorption of the PMS molecule onto the pyridinic nitrogen sites [14]. Furthermore, electron transfer is facilitated by the extended π-conjugated structure inherent in pyridinic nitrogen-doped carbon materials, thereby enhancing the catalyst’s activation efficiency towards PMS [15,16]. Efficient activation of PMS and degradation of organic pollutants are achieved by nitrogen-doped carbon materials during the PMS activation process. However, poisoning reactions readily occur at Lewis basic active sites, leading to a reduction in catalytic activity [17]. Notably, regeneration of catalytic activity is achieved through high-temperature treatment [12,18,19]. The reversible adsorption of surface hydroxyl groups is widely reported in oxygen reduction and other catalytic systems, whereas this behavior is rarely documented for carbon-based catalysts in PMS activation processes. Furthermore, the recent literature on catalyst deactivation and regeneration during PMS activation remains largely focused on phenomenological observations, while the underlying mechanisms driving these phenomena are insufficiently explored [20,21,22].

Methylene blue was identified as a representative recalcitrant cationic dye, which possessed a stable structure and demonstrated well-defined ecotoxicity and environmental risks. Consequently, a nitrogen-doped carbon material catalyst featuring Lewis basic sites was prepared in this study for the activation of PMS to treat MB. The goal of the research was to systematically investigate the molecular mechanisms underlying the deactivation and reactivation pathways of carbon-based Lewis basic sites. The deactivation and recovery evolutions of catalysis sites were analyzed through combined XPS, FTIR, and TPD techniques. The carbon atoms adjacent to pyridinic nitrogen were identified as the Lewis basic active sites. Deactivation of Lewis basic sites stemmed from hydroxyl OH(ads) adsorption during PMS activation, which binds to the sites and causes inactivation. Both high-temperature treatment under inert gas protection and room-temperature treatment with NaBH_4_ effectively removed OH(ads) attached to the basic sites, thereby restoring catalytic activity. The OH(ads)-induced deactivation of basic sites and subsequent recovery mechanism demonstrated remarkable universality in the degradation of various organic pollutants, including antibiotics (SMZ), dyes (RhB), pesticides (MCPA), and endocrine disruptors (BPA).

## 2. Experimental Section

### 2.1. Materials

All chemicals referenced in Text S1 were of analytical grade or higher and used as received. Ultra-pure water (Milli-Q, 18.2 MΩ·cm at 25 °C, Merck KGaA, Darmstadt, Germany) was employed for all aqueous solution preparations throughout the experimental procedures.

### 2.2. Preparation and Characterization of Catalysts

Nitrogen-doped biochar catalysts (denoted as NC-T, where T represented pyrolysis temperature) were synthesized via argon-protected pyrolysis using heavy oil and melamine as carbon and nitrogen precursors, respectively, with magnesium citrate acting as a porogenic template. Three thermal variants (NC-800, NC-900, and NC-1000) were prepared alongside non-doped biochar (BC) for comparative assessment. The physicochemical properties of the resultant catalysts were systematically characterized through multi-modal analytical techniques. Comprehensive synthetic protocols and characterization methodologies were detailed in Texts S2 and S3.

### 2.3. Experimental Procedure

Methylene blue (MB) degradation experiments were conducted in conical flasks, each containing 50 mL of MB solution (50 mg/L), PMS (3 mM), and catalyst (0.3 g/L). The mixtures were stirred continuously at 450 revolutions per minute using magnetic stirring at room temperature. Sampling was initiated 3 min after the reaction commenced, with subsequent samples collected at 5 min intervals throughout the 30 min reaction period. All samples were immediately filtered through 0.45 micron membranes and quenched with methanol (MeOH) to terminate radical reactions prior to analysis. Active species were identified via scavenging experiments employing furfuryl alcohol (FFA), tert-butanol (TBA), p-benzoquinone (BQ), or ethanol (EtOH). Parameter-dependent performance was evaluated by systematically varying reaction conditions to assess MB degradation kinetics. For cycling experiments, catalysts were recovered, washed three times with ethanol followed by water, and dried under vacuum at 60 °C for 48 h. All experiments were performed in triplicate to ensure reproducibility. More details of the experiment are available in the Supplementary Materials.

## 3. Results and Discussion

### 3.1. Catalysts Characterization

**Figure 1:**
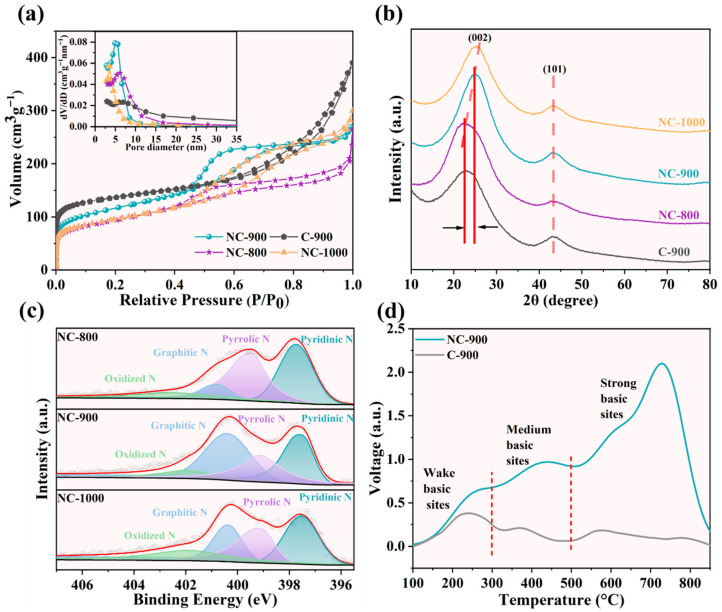
(**a**) N_2_ adsorption–desorption isotherm and pore size distribution of C-900, NC-800, NC-900, and NC-1000. (**b**) XRD spectra of C-900, NC-800, NC-900, and NC-1000 catalysts. (**c**) N1s spectra of C-900, NC-800, NC-900, and NC-1000 catalysts. (**d**) The CO_2_-TPD curves of C-900, NC-800, NC-900 and NC-1000 catalysts. Among them, C-900 is a carbon catalyst synthesized at 900 °C, while NC-800, NC-900, and NC-1000 are nitrogen-doped carbon catalysts prepared at 800 °C, 900 °C, and 1000 °C, respectively.

SEM and TEM images of C-900, NC-800, NC-900, and NC-1000 were presented in Figure S1. The images revealed that C-900 possessed a smooth surface morphology, whereas the nitrogen-doped catalysts exhibited marked wrinkles and pores, which were attributed to gas evolution during the pyrolysis of melamine [23]. Progressive elevation of pyrolysis temperature induced microporous structure collapse, resulting in enlarged macropore formation. Corresponding Brunauer–Emmett–Teller (BET) surface areas and N_2_ adsorption–desorption curves were summarized in Table S1 and Figure 1a. The NC-900 catalyst demonstrated a specific surface area of 402.6 m^2^/g via BET analysis, lower than that of C-900 (570.4 m^2^/g). This reduction likely originated from nitrogen-functional groups (e.g., pyridinic-N and pyrrolic-N) occupying pore volumes. Following nitrogen doping, the adsorption isotherm of the sample exhibited combined features of Type I and Type IV (Figure 1a), which was typical of a micro-mesoporous hybrid structure [24]. The pronounced N_2_ adsorption at low relative pressures confirmed the existence of a microporous structure in the material. As shown in Table S1, the micropore-specific surface area increased significantly. This enhancement was attributed to the vaporization of melamine during pyrolysis, which acted as a gasifying agent to generate additional micropores [25]. At higher relative pressures, a distinct hysteresis loop appeared, with the desorption branch lying above the adsorption branch. This hysteresis was ascribed to capillary condensation within the mesoporous structure [26]. Energy-dispersive X-ray spectroscopy (EDS) mapping confirmed the homogeneous dispersion of C, N, and O elements (Figure S1d) throughout the NC catalysts.

The X-ray diffraction (XRD) patterns of C-900, NC-800, NC-900, and NC-1000 were displayed in Figure 1b. The XRD profile of C-900 exhibited two broad diffraction peaks at approximately 2θ = 23° and 43.5°, corresponding to the (002) and (101) crystallographic planes of the carbon materials, respectively [27]. The broad peak observed at 23° signified the characteristic amorphous carbon structure, confirming the non-crystalline nature of C-900. Upon nitrogen doping, this characteristic peak shifted toward higher angles, indicating structural alterations within the carbon matrix. Furthermore, NC-900 demonstrated significantly enhanced crystallographic peak intensity compared to C-900. The elevated peak intensities reflected the higher graphitization degree, revealing that nitrogen doping promoted graphitic ordering in the carbon frameworks [28]. Enhanced graphitization facilitated interfacial electron transfer during PMS activation [29]. This confirmed the generation of additional defect sites, and the pyrolysis temperature variations further modulated defect concentrations in the catalysts.

Elemental analysis results (Table S2) confirmed successful nitrogen incorporation into the carbon matrix via melamine introduction, with progressive nitrogen content reduction observed upon increasing pyrolysis temperatures. The XPS was employed to probe elemental composition and chemical configurations. The survey spectra analysis (Figure S2) demonstrated three distinct peaks corresponding to C, O, and N for NC-800, NC-900, and NC-1000, contrasting with only C and O peaks in C-900, verifying effective N-doping. Quantitatively, the nitrogen contents of 14.29, 6.50, and 4.22 at% were determined for NC-800, NC-900, and NC-1000, respectively, aligning with the elemental analysis trends. The gradual nitrogen loss from 800 to 1000 °C indicated thermal decomposition of unstable nitrogen configurations [30]. The deconvolution of high-resolution N1s spectra (Figure 1c) revealed four constituent peaks: pyridinic N (397.54 eV), pyrrolic N (399.02 eV), graphitic N (400.36 eV), and N-oxides (401.91 eV) [31]. The variation chart of nitrogen components (Figures S3 and S4) showed that thermally unstable pyrrolic N declined from 39% at 800 °C to 27% at 1000 °C, while the moderately stable pyridinic N decreased from 37% (800 °C) to 28% (900 °C) before stabilizing at 1000 °C. The nitrogen doping content also decreased progressively with increasing pyrolysis temperature. Conversely, the thermally robust graphitic N increased from 12% to 26% across this temperature range, consistent with the survey spectra observations and confirming thermal decomposition mechanisms of the nitrogen functionalities. The CO_2_ adsorption experiment revealed a distinct peak intensity in the curve between 600 °C and 800 °C following nitrogen doping (Figure 1d). This indicated that nitrogen doping generated Lewis basic sites [32].

### 3.2. Evaluation of Catalytic Activity

**Figure 2:**
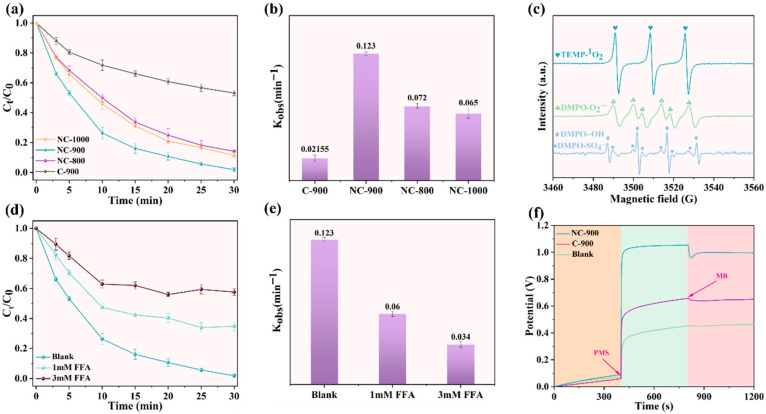
(**a**) The removal rate of MB in the C-900, NC-800, NC-900, and NC-1000/PMS systems. (**b**) The pseudo-first-order kinetic reaction rate constants for MB removal in the C-900, NC-800, NC-900, and NC-1000/PMS systems. (**c**) DMPO and TEMP trapped EPR spectra in the NC-900/PMS system. (**d**) In the NC-900/PMS system, the effects of ^1^O_2_ radical scavengers on MB degradation (1mMFFA and 3mMFFA). And (**e**) pseudo-first-order kinetic curves. Reaction conditions: [catalysts] = 15 mg, [PMS] = 3 mM, [MB] = 50 mg/L, T = 25 °C. (**f**) Open-circuit voltage in the C-900 and NC-900/PMS systems changes with the addition of PMS and MB, respectively.

The adsorption capacities of C-900, NC-800, NC-900, and NC-1000 for methylene blue (MB) were evaluated under identical conditions (Figure S5). All catalysts exhibited similar adsorption performance, achieving only 10% MB removal within 30 min. This demonstrated that the contribution of adsorption to the overall removal process was negligible. When PMS alone was applied, merely 15% MB removal was attained after 30 min, revealing its intrinsically slow degradation kinetics. This limited efficiency necessitated catalytic activation to achieve rapid pollutant decomposition. The catalytic performance of the catalysts was evaluated by the rate of MB degradation activated by PMS. The catalytic degradation experiments were conducted under identical conditions for different catalysts. The results demonstrated that the NC-900/PMS system achieved near-complete degradation of MB (97.2%) within 30 min, whereas only 47% removal efficiency was observed for the C-900/PMS system (Boulder, CO, USA). This indicated that the Lewis basic sites enhanced the capability of carbon-based catalysts to activate PMS. The doping of nitrogen was demonstrated to induce a marked elevation in Lewis basic sites within the carbon materials (Figure 1d), which consequently imparted significantly enhanced degradation efficacy and an elevated pseudo-first-order reaction rate constant to nitrogen-doped carbon materials (Figure 2a,b).

### 3.3. Identification of Reactive Species

To further characterize the reactive oxygen species (ROS) generated in the NC-900/PMS system, electron paramagnetic resonance (EPR) spectroscopy was employed using DMPO and TEMP as spin-trapping agents. Figure 2c showed discernible DMPO-•OH, DMPO-SO_4_^•−^, DMPO-O_2_^−^, and TEMP−^1^O_2_ signals were detected at 1 min and 3 min (Figure S6) after the addition of NC-900, indicating that SO_4_^•−^, •OH, O_2_^−^, and ^1^O_2_ were sequentially generated during the reaction [12,33]. Multiple scavengers were employed in a series of quenching experiments to identify which reactive oxygen species (ROS) were involved in the degradation of MB [34]. The TBA exhibited preferential reactivity toward the •OH due to its reaction rate constant with the •OH exceeding that with the SO_4_^•−^ by several orders of magnitude [35]. The MB degradation efficiency decreased drastically from 100% to 40% upon quenching the ^1^O_2_, whereas quenching other radicals reduced efficiency by only 20%, indicating their limited contributions (Figure 2d,e and Figures S7–S9). Therefore, PMS could be activated by the Lewis basic sites to generate the ^1^O_2_, which also served as the predominant ROS in the NC-900/PMS system [36].

In addition to the ^1^O_2_ pathway, the electron transfer pathway was identified as another non-radical pathway operating within the biochar-activated PMS system [37]. The NC-900 electrode was utilized as the working electrode to investigate its electron transfer pathway capability through linear sweep voltammetry (LSV) and open-circuit potential (OCP) measurements. The LSV curve representation (Figure S10) shows that a marked increase in current intensity was observed when PMS coexisted with the catalyst compared to the system containing PMS alone, indicating the occurrence of electron transfer within the PMS/NC-900 system. Furthermore, the OCP of the NC-900 electrode exhibits a significant rise following PMS introduction (Figure 2f), with a subsequent potential decrease upon the MB addition. This result suggests that a complex was formed between NC-900 and PMS, wherein the MB was consumed as an electron acceptor via electron transfer in the presence of MB [38]. The i–t curve indicates (Figure S11) electron transfer between the catalyst and PMS, and the nitrogen doping enhances the electron transfer capability. Galvanically coupled oxidation system (GOS) and electrochemical impedance spectroscopy (EIS) Nyquist plot further evidence the existence of electron transfer (Figures S12 and S13) between the catalyst and PMS [39,40], while the nitrogen doping is demonstrated to enhance the electron transfer capability of the catalyst.

### 3.4. Determination of the Active Sites

**Figure 3:**
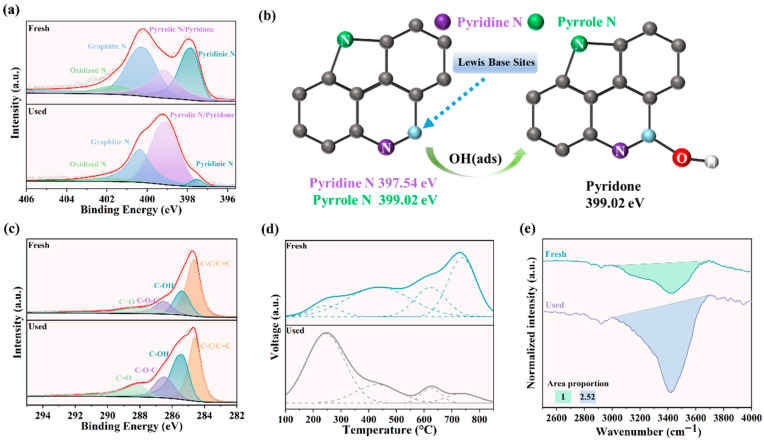
Material characterization of fresh catalyst NC-900 and used catalyst NC-900. (**a**) XPS spectra of N1s. (**b**) The schematic representation illustrated that OH(ads) bonded to Lewis basic sites adjacent to pyridinic nitrogen, resulting in an increased binding energy of pyridinic nitrogen in XPS analysis. (**c**) XPS spectra of C1s. (**d**) The CO_2_-TPD curve during the process ranges from 100 °C to 850 °C. (**e**) The comparison diagram of the hydroxyl peak in the FTIR spectrum.

Through XPS analysis of the recovered catalyst in the NC-900/PMS system, changes in its surface chemical composition were examined. The N1s spectrum (Figure 3a) demonstrated that the pyridinic nitrogen peak of the recovered sample exhibited a significant decrease, declining from 27.91% to 5.56%. In contrast, the pyrrolic nitrogen peak displayed a substantial increase, rising from 23.51% to 59.89% (Figure S14). The experimental evidence indicated a substantial depletion of the pyridinic nitrogen species throughout the PMS activation process. The mechanistic origin of the observed concomitant rise in pyrrolic nitrogen content, nevertheless, warrants further investigation. Previous studies demonstrated that chemisorption of OH(ads) on carbon atoms adjacent to pyridinic nitrogen sites increased the binding energy of pyridinic nitrogen to 399 eV in XPS spectra [41]. The altered binding energy closely approached that of pyrrolic nitrogen, resulting in a pseudomorphic enhancement of the pyrrolic nitrogen characteristic peak in N1s spectra. Therefore, the decrease in the pyridinic nitrogen peak after PMS activation was not due to the conversion of the pyridinic nitrogen to the pyrrolic nitrogen (Figure 3b), but rather arose from the pyridone inducing a spectral shift [41,42,43]. Pyridinic nitrogen doping rendered adjacent carbon atoms Lewis basic sites [44]. The elevated oxygen content in the XPS survey scan of the catalyst (Figure S15) could be attributed to the presence of pyridone species. Based on the CO_2_-TPD analysis of the samples before and after the reaction, a significant attenuation of strong basic sites and a marked enhancement of weak basic sites were observed in NC-900, indicating the involvement of Lewis basic sites in the catalytic process [45]. Consequently, in the nitrogen-doped carbon catalysts, the carbon atoms adjacent to pyridinic nitrogen exhibited significant PMS activation capability. Moreover, the nitrogen atoms at the pyridinic nitrogen sites were poisoned [46,47]. The results (Figure S16) showed that the catalytic activity of the catalyst did not change, which indicated that the active sites of the catalyst were not the pyridinic nitrogen atoms but rather the adjacent carbon atoms with Lewis basicity.

To verify this conclusion, we conducted an in-depth analysis of the C1s and O1s spectra of the recovered catalyst through XPS. The O1s (Figure S17) spectrum deconvolved into three peaks: 530.8 eV (carbonyl C=O), 532.0 eV (hydroxyl/carbon–oxygen bonds in aliphatic chains: C–OH and C–O–C), and 533.3 eV (phenolic C–OH in aromatic systems) [48]. The C1s spectra (Figure 3c) deconvolution revealed four distinct chemical states at 284.6 eV (C–C/C=C), 285.4 eV (C–OH), 286.5 eV (C–O–C), and 288.1 eV (C=O), corresponding to specific carbon bond configurations [49]. The post-catalysis O1s spectral analysis revealed increased hydroxyl groups associated with the aromatic carbon frameworks (C–OH) alongside decreased hydroxyl/ether group density bound to the aliphatic chains (C–OH/C–O–C). This indicated that the hydroxyl groups were covalently bonded to the aromatic carbon after activated PMS oxidation, corroborated by elevated C–OH relative content in the C1s spectra. The hydroxyl groups attached to aromatic rings were categorized as in the TPD analysis (Figure 3d), corresponding to the markedly increased intensity of the weak basic sites observed after the reaction [50,51]. Comparative analysis of FTIR spectra (Figure 3e) for the catalyst samples before and after reaction revealed a marked intensification of the O–H stretching vibration band at 3200–3600 cm^−1^ in the post-reaction sample, with a quantitative ratio of 1:2.52 determined. The result constituted the most conclusive evidence that the attachment of hydroxyl groups to the Lewis basic sites occurred during the PMS activation process. The Raman spectra further revealed a reduction in the defect density associated with nitrogen doping (Figure S18). Critically, all samples underwent identical rigorous vacuum drying at 60 °C for 48 h to eliminate interference from moisture in hydroxyl group detection. This confirmed that the observed enhancement originated from genuine surface chemical modification, not artifacts caused by residual water. Consequently, the nitrogen configuration changes exhibited a correlation with the hydroxyl groups bound to the aromatic carbon skeleton. This observation confirmed that the chemisorption occurred between Lewis basic sites adjacent to pyridinic nitrogen and adsorbed hydroxyl species, OH(ads), following the catalytic reaction (Figure 3e). Subsequent to OH(ads) attachment, the catalyst exhibited pronounced activity attenuation, which further substantiated that the Lewis basic sites functioned as active centers for PMS activation.

### 3.5. Catalyst Activation Recovery

**Figure 4:**
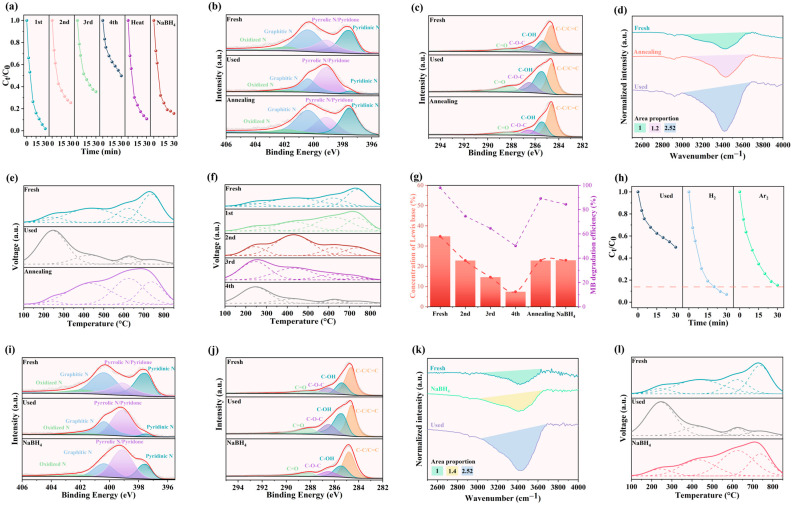
(**a**) Stability tests of pristine NC-900 samples for treating MB in solution. (**b**) XPS spectra of N1s and (**c**) C1s (XPS characterization of fresh catalyst NC-900, used catalyst NC-900, and heat-treatment-regenerated catalyst NC-900). (**d**) The comparison diagram of the hydroxyl peak in the FTIR spectrum. And (**e**) the CO_2_-TPD curve during the process ranging from 100 °C to 850 °C (fresh catalyst NC-900, used catalyst NC-900 and thermal-treatment-regenerated catalyst NC-900). (**f**) CO_2_-TPD curve diagram of the NC-900 sample during the cycling process. (**g**) Comparison diagram of Lewis basic site and catalytic efficiency. (**h**) The removal rate of MB in the used catalyst NC-900 and the catalyst NC-900 prepared by thermal regeneration under hydrogen and argon protection. (**i**) XPS spectra of N1s and (**j**) C1s (XPS characterization of fresh catalyst NC-900, used catalyst NC-900 and heat-treatment-regenerated catalyst NC-900). (**k**) The comparison diagram of the hydroxyl peak in the FTIR spectrum. and (**l**) the CO_2_-TPD curve during the process ranging from 100 °C to 850 °C. (Among them, “Fresh” refers to the unused catalyst NC-900, “Used” refers to the catalyst NC-900 that has been recycled and processed, “Annealing” refers to the catalyst NC-900 that has undergone thermal-treatment regeneration after recycling, and “NaBH_4_” refers to the catalyst NC-900 that has been regenerated by using sodium borohydride after recycling and use).

The above studies demonstrated that the deactivation mechanism of nitrogen-doped carbon materials originated from chemisorption of the OH(ads) onto Lewis basic sites during PMS activation, thereby compromising their catalytic activity. Consequently, the critical factor for regenerating the catalyst’s activity resided in the removal of OH(ads) attached during the reaction process. Therefore, the regeneration method involving heat treatment under an inert atmosphere was employed for the recovered catalysts to restore their activity. The catalyst deactivation and regenerated performance were illustrated in Figure 4a, revealing that the pristine NC-900 achieved nearly complete MB degradation within 30 min, whereas its efficiency declined to 80% after the second cycle, with further reduction to 60% and 50% following the third and fourth cycles, respectively. Following four catalytic cycles, the activity of NC-900 converged with that of C-900. The catalytic enhancement attributable to basic site introduction diminished by 93.28%, with these sites exhibiting near-total deactivation. Notably, calcination at 900 °C for 1 h under an argon atmosphere restored the degradation efficiency to 90%.

To elucidate the mechanism of catalytic activity regeneration, XPS and FTIR spectroscopy were employed to characterize the surface chemical composition of the regenerated catalysts, while CO_2_-TPD analysis was conducted to quantify the density of Lewis basic sites. The N 1s spectra of thermally regenerated catalysts (Figure 4b) demonstrated an increase in the pyridinic nitrogen content from 5.56% to 31.35%, approaching the pristine catalyst level, whereas the pyrrolic nitrogen decreased from 59.89% to 23.47%, indicating restoration of the nitrogen chemical states to the unused catalyst level after high-temperature treatment. Concurrently, the C 1s and O 1s spectra (Figure 4c and Figure S19) revealed a reduction in the peak intensity of C–OH groups on aromatic carbon rings. These XPS results suggested that activity recovery originated from desorption of the OH(ads) species bound to Lewis basic sites, shifting the binding energy of hydroxyl-associated pyridinic groups from 399 eV to 397.5 eV, thereby accounting for the decrease in the pyrrolic nitrogen and increase in the pyridinic nitrogen content. The FTIR spectra (Figure 4d) confirmed a significant attenuation of the hydroxyl peak following heat treatment, with the reduction in peak area ratio from 2.52 to 1.2 [52]. The Raman spectroscopy (Figure S20) revealed that the defect density induced by nitrogen doping in the catalyst was substantially restored to its initial level after high-temperature treatment [53]. Additionally, the CO_2_-TPD (Figure 4e) profiles revealed a significant shift from weak to strong basic sites in the regenerated Lewis basic sites, thereby confirming the desorption of OH(ads) from carbon atoms adjacent to pyridinic nitrogen sites observed in the XPS spectra. This revealed that the high-temperature-induced desorption of the OH(ads) species bound to Lewis basic sites.

The TPD analysis performed on the catalyst regenerated through four consecutive cycles revealed a progressive transformation of strong basic sites into weak basic sites with increasing cycle numbers (Figure 4f). The concentration of strong basic sites progressively declined from 34.79% to 7.44% after four reaction cycles but was restored to 22.85% following regeneration treatment. A significant correlation was observed between the variation in the aforementioned Lewis basic sites and the change in catalytic efficiency (Figure 4g), thereby demonstrating the pivotal role of pyridinic nitrogen-doped basic sites in the catalyst’s ability to activate PMS [54]. Additionally, high temperatures at 700 °C under pure argon and argon–hydrogen mixed gas atmospheres were designed to regenerate catalyst activity. Annealing at 700 °C under argon restored approximately 85% of the degradation efficiency (Figure 4h), whereas treatment under the argon–hydrogen mixed gas atmosphere achieved near-complete restoration of activity. This demonstrated that the hydrogen-mediated reduction at elevated temperatures significantly enhanced the regeneration of deactivated catalysts.

Therefore, NaBH_4_ solution was employed to reduce the OH(ads) on the surface generated during the catalytic process. The NaBH_4_ solution treatment recovered exceeding eighty-five percent catalytic efficiency. The characterization analysis of the NaBH_4_-treated catalyst similarly revealed spectral features that were strikingly similar to those of the thermally regenerated catalyst, with the pyridinic nitrogen content increasing from 5.56% to 16.66% (Figure 4i) while the pyrrolic nitrogen content decreased from 59.89% to 47.54%. Comparative analysis of the C 1s (Figure 4j) and O 1s (Figure S21) spectra indicated a reduction in signals corresponding to C–OH groups. FTIR spectroscopy (Figure 4k) revealed a significant attenuation of the hydroxyl peak ratio from 2.52 to 1.40. A pronounced negative correlation was observed between the variation in hydroxyl peak intensity and alterations in the catalyst’s activation capability (Figure S22), which indicated that increased hydroxyl groups substantially suppressed the catalyst’s activity. FTIR spectra revealed a significant attenuation of the hydroxyl peak, while CO_2_-TPD spectra confirmed a substantial increase in the density of Lewis strong basic sites (Figure 4l), demonstrating that the poisoning effect of adsorbed OH(ads) onto Lewis basic sites was effectively reversed through desorption during NaBH_4_ treatment, thereby restoring Lewis basicity, which aligned with the recovery of over 85% of the catalytic efficiency. The concentration of C–OH increased from 24.53% to 35.40% during the reaction process but was decreased to approximately 25.73% following regeneration treatment (Figure S23). The variation in C–OH content exhibited a pronounced negative correlation with the catalytic activity of the catalyst, which indicated that the C–OH sites could compromise the catalyst’s capacity to activate PMS, further corroborating that the carbon atoms adjacent to pyridinic nitrogen constituted the active sites.

During MB degradation catalyzed by NC-900, ultraviolet-visible (UV-Vis) spectral evolution exhibited a distinct blue-shifting of the absorption maximum (Figure S24). In contrast, no spectral displacement occurred with the C-900 catalyst, a phenomenon attributed to Lewis basic sites activating PMS to accelerate MB degradation via radical-mediated pathways [55]. It was noteworthy that after four catalytic cycles, the blue shift exhibited by NC-900 significantly attenuated, and its spectral profile gradually approached that of C-900. The attenuation of this blue shift demonstrated a positive correlation with the Lewis basic content, which directly indicated the involvement of the Lewis basic sites in altering the MB conjugated structure. Furthermore, following high-temperature treatment for active site regeneration, the intensity of the blue shift observed during the degradation process recovered significantly. This recovery aligned precisely with the restoration of Lewis basic sites observed in TPD analysis, confirming the successful regeneration of the Lewis basic sites that were consumed during the reaction cycles and were crucial for the catalytic reaction.

### 3.6. Reaction Mechanism

**Figure 5:**
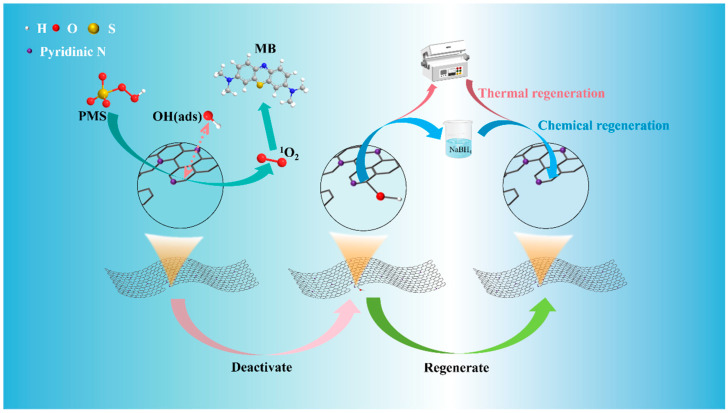
The schematic diagram illustrating the molecular mechanism of the inactivation and reactivation process of carbon-based Lewis basic sites during the catalytic PMS process.

Based on the above analysis, we propose that in the NC-900/PMS system, the carbon atoms adjacent to the pyridine nitrogen function as Lewis basic sites and can serve as active sites for PMS (Figure 5). The Lewis basic sites exhibit strong affinity for PMS, tending to adsorb onto the O1 site of PMS, thereby promoting the dissociation of the peroxy bond (O–O) within PMS. This dissociation process generates reactive species such as ^1^O_2_ and adsorbed oxygen atoms O(ads), enabling the oxidative degradation of MB. The adsorbed oxygen atom O(ads) reacts with water and electrons to form an adsorbed hydroxyl group OH(ads). The OH(ads) attaches to the carbon atom adjacent to the pyridine nitrogen atom, thereby weakening the adsorption affinity of the Lewis basic site for PMS and leading to its deactivation. The OH(ads) attached to the carbon atom can be removed through high-temperature treatment or NaBH_4_ reduction treatment, thereby restoring the activity of the Lewis basic site.

### 3.7. The Influence of Reaction Parameters

The effect of the initial solution pH on MB degradation was illustrated in Figure S25. Degradation efficiencies reached 70%, 74%, 90%, and 100% at 10 min under pH 3, 6, 8, and 10, respectively. Notably, the acidic conditions (pH 3–6) exerted minimal influence on degradation kinetics, with the rate constant decreasing marginally from 0.123 min^−1^ to 0.118 min^−1^. Conversely, the alkaline conditions markedly enhanced degradation kinetics: at pH = 8, k increased to 0.185 min^−1^, while at pH 10, a sharp kinetic acceleration occurred (k = 0.356 min^−1^). This phenomenon suggested preferential generation of SO_5_^•−^ radicals under alkaline conditions, facilitating enhanced ^1^O_2_ production and consequent rapid MB degradation. Furthermore, the cationic nature of MB and the negatively charged surface of NC-900 at alkaline pH intensified electrostatic attraction, promoting rapid contact between MB and oxidative species, thereby accelerating degradation efficiency [56,57].

Furthermore, the influence of inorganic anions in real wastewater matrices on MB degradation warranted attention (Figure S26). As demonstrated, introducing 10 mM Cl^−^ increased the rate constant (k) from 0.123 min^−1^ to 0.216 min^−1^, attributable to chlorine reactive species generated via Cl^−^-PMS reactions that exhibited high selectivity toward MB. Similarly, 10 mM CO_3_^2−^ enhanced degradation kinetics (k = 0.153 min^−1^ vs. 0.123 min^−1^ control) through carbonate radical (CO_3_^•−^) formation and subsequent generation of lower-oxidation-state species. Conversely, 10 mM SO_4_^2−^ and NO_3_^−^ marginally suppressed degradation efficiency, reducing k to 0.102 min^−1^ and 0.094 min^−1^, respectively, due to competitive consumption of oxidative species. Critically, near-complete MB degradation persisted despite the anion interference, demonstrating exceptional ion interference resistance in the NC-900/PMS system. This characteristic was regarded as a significant advantage of the non-radical oxidation pathway. This robustness underscored NC-900’s adaptability to complex environmental matrices. The influence of various parameters within the NC-900/PMS system on degradation efficiency (Figures S27 and S28) was also investigated.

### 3.8. Possible Degradation Pathways and Toxicological Analysis of MB

The MB molecule was attacked by radical and non-radical species generated from NC-900-activated PMS, leading to its fragmentation into unstable intermediates that were gradually oxidized and ultimately transformed into harmless end-products. Thus, primary intermediates were identified via LC-MS and GC-MS analyses, enabling the elucidation of their specific degradation pathways. The results presented in Figure S29 revealed that intermediate P1 was formed via demethylation of the MB molecule. Meanwhile, intermediates P2 and P3 were generated via complete demethylation and oxidation of the amino group. These intermediates underwent further oxidative cleavage, yielding intermediates P4, P5, and P6–P9, which were subsequently mineralized into harmless end-products such as CO_2_ and H_2_O.

The potential ecotoxicity to three aquatic organisms (fish, daphnia, and green algae) of MB and nine transformation products generated during degradation was assessed using the ECOSAR program. As shown in Figure S31 and Table S3, the acute and chronic toxicity of MB’s transformation products to these three aquatic organisms was assessed based on GHS classification standards. The results indicated that all products exhibited lower toxicity than MB, and the majority were classified as non-toxic. The final products were further mineralized into carbon dioxide and water via ROS-mediated oxidation, thereby reducing toxicity to acceptable levels for aquatic organisms.

### 3.9. Universality of Active Site Deactivation and Regeneration Mechanisms

**Figure 6:**
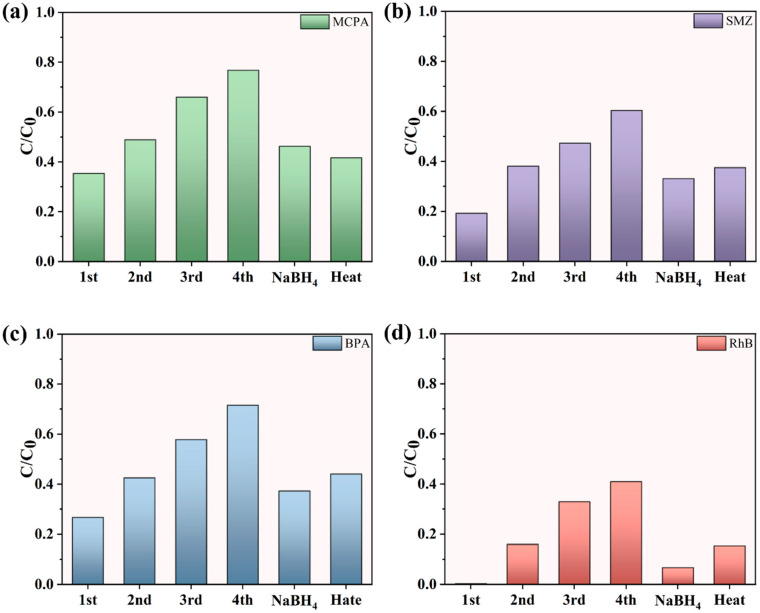
The removal rates of different pollutants during the cycling process and the regeneration process of the catalyst NC-900. (**a**) the 2-methyl-4-chlorophenoxyacetic acid (MCPA). (**b**) the antibiotic sulfamethazine (SMZ). (**c**) the endocrine disruptor bisphenol A (BPA). (**d**) the dye rhodamine B (RhB).

In addition to the utilization of MB as a model contaminant, the degradation efficiencies of various other pollutants were systematically investigated, along with the universal phenomenon of active site poisoning and regeneration during the degradation processes. Four representative contaminants, including the pesticide 4-chloro-2-methylphenoxyacetic acid (MCPA), the antibiotic sulfamethazine (SMZ), the dye rhodamine B (RhB), and the endocrine disruptor bisphenol A (BPA), were selected for evaluation to determine their activity attenuation over four consecutive catalytic cycles. Subsequently, regeneration was achieved through NaBH_4_ treatment and high temperature under an argon atmosphere. The results (Figure 6a–d) demonstrated that all four pollutants exhibited varying degrees of activity decay during cycling, but the activity recovery rates after regeneration exceeded 80%. This confirmed that the poisoning and subsequent reactivation of Lewis basic sites during PMS activation for pollutant degradation constituted a universal mechanism rather than an isolated phenomenon.

### 3.10. Limitations and Future Work

Although the present experiment demonstrated that the deactivated catalyst could be regenerated by removing adsorbed hydroxyl groups from carbon atoms adjacent to pyridinic nitrogen. However, according to the conclusions derived from this study, future investigations still require electronic modulation to mitigate the interaction between Lewis basic sites of the catalyst and adsorbed hydroxyl groups, thereby extending the cycling stability of the catalyst and promoting practical applications of nitrogen-doped carbon catalysts.

## 4. Conclusions

This work explored the composition transformations of Lewis basic carbon atoms adjacent to pyridinic nitrogen during advanced oxidation processes. The experimental results demonstrated that the carbon atoms with Lewis basicity acted as prominent catalytic sites for PMS activation. The reversible chemisorption and desorption of hydroxyl groups on the carbon atom were identified as the core mechanism governing catalyst deactivation and regeneration. The C–OH content was increased from 25.53% to 35.4% throughout the catalytic process, while the degradation capability of NC-900 was reduced from 98.07% to 50.17%. Following regeneration treatment, the C–OH content of NC-900 was reduced to 25.73%, while its catalytic activity was restored to 89.22%. A reversible variation in the content of Lewis strong basic sites was similarly observed from 34.79% to 7.44% and subsequently recovered to 22.85%. Notably, the proposed process for the regenerated catalyst demonstrated applicability across different contaminant categories, including antibiotic and pesticide systems. This work provided theoretical foundations for renewable catalysts in advanced oxidation processes. This approach established a new theoretical foundation for designing robust, tunable catalysts with regeneration potential for a wide range of advanced oxidation processes.

## Data Availability

The original contributions presented in this study are included in the article/Supplementary Materials. Further inquiries can be directed to the corresponding authors.

## Supplementary Materials

The following supporting information can be downloaded at: https://www.mdpi.com/article/10.3390/ma19081589/s1.

## References

[1] Ma H., Yu L., Yang L., Yao Y., Shen G., Wang Y., Li B., Meng J., Miao M., Zhi C. (2024). Graphene oxide composites for dye removal in textile, printing and dyeing wastewaters: A review. Environ. Chem. Lett..

[2] Sun C., Li M., Wang Q., Liu R., Li M., Chen F., Xu S., Wang G. (2025). Enhanced degradation of rhodamine B by UV/Co_3_O_4_@BC/PMS system: Performance and mechanism. J. Water Process Eng..

[3] Liu K., Guo X., Liu Y., Wang X., Wang J., Wang X., Zhang L., Zhu Y., Yang D. (2026). White LED driven {010}-faceted BiVO4 mediated electron transfer enables efficient peroxymonosulfate activation for norfloxacin degradation. Adv. Powder Mater..

[4] Meng H., Zhou J., Zhang Y., Cui J., Chen Y., Zhong W., Chen Y., Jia C.Q. (2025). Single-atom Co-N3 sites induce peroxymonosulfate activation for acetaminophen degradation via nearly 100% internal electron transfer process. Appl. Catal. B Environ. Energy.

[5] Chen T., Zhang G., Sun H., Hua Y., Yang S., Zhou D., Di H., Xiong Y., Hou S., Xu H. (2025). Robust Fe-N_4_-C_6_O_2_ single atom sites for efficient PMS activation and enhanced Fe^IV^ = O reactivity. Nat. Commun..

[6] Sheng B., Huang X., Zhao Q., Zhang Z., Duan R., Zhang J., Chen C., Zhao J., Sheng H. (2025). Discriminative Peroxymonosulfate Activation on Iron Carbides for Redox-Neutral Singlet Oxygen Generation. Angew. Chem. Int. Ed. Engl..

[7] Li Z., Zhang X., Xue M., Wei M., Chen S., Lu Q., Guo E., Han X., Si C. (2025). Enhanced peroxymonosulfate activation in organic degradation by modulating cationic doping of Ba_x_Sr_1-x_Co_y_Fe_1-y_O_3-δ_ perovskites: DFT calculations and mechanism study. Appl. Catal. B Environ. Energy.

[8] Wang F., Gao Y., Fu H., Liu S.-S., Wei Y., Wang P., Zhao C., Wang J.-F., Wang C.-C. (2023). Almost 100% electron transfer regime over Fe−Co dual-atom catalyst toward pollutants removal: Regulation of peroxymonosulfate adsorption mode. Appl. Catal. B.

[9] Liang X., Zhao Y., Liu B., Li J., Cui L., Wang C., Yang Q. (2024). Phosphorus doped magnetic biochar activated PMS for effective degradation of pesticide in water: Targeted regulation of interfacial charge transfer by phosphorus doping. Sep. Purif. Technol..

[10] Wang Y., Zhang Z., Yin Z., Liu Z., Liu Y., Yang Z., Yang W. (2022). Adsorption and catalysis of peroxymonosulfate on carbocatalysts for phenol degradation: The role of pyrrolic-nitrogen. Appl. Catal. B.

[11] Yang M., Liu W., Liu Q., Chen Z., Cao J., Luo J., Xing M. (2025). Constrained Heterogeneous CoFe_2_O_4_/ZnO/PMS Fenton-Like System for Industrial Wastewater Remediation with Recyclability and Zero Metal Loss. Angew. Chem. Int. Ed. Engl..

[12] Di L., Wang T., Lu Q., Lu J., Zhang Y., Zhou Y., Zhou Y. (2024). Efficient PMS activation toward degradation of bisphenol A by metal-free nitrogen-doped hollow carbon spheres. Sep. Purif. Technol..

[13] Zou L., Yang K., Hu Y., Guo X., Li X., Lv Y., Liu Y., Ye X., Lin C., Liu M. (2024). Nitrogen-doped cobalt oxide via atmospheric modulation activates PMS for rapid degradation of organic pollutants: Role of nitrogen fraction on sulfapyridine elimination. J. Water Process Eng..

[14] Yang M., Wang W., Ma H., Chen L., Ma H., Shi F. (2025). Efficient tetracycline hydrochloride degradation via peroxymonosulfate activation by N doped coagulated sludge based biochar: Insights on the nonradical pathway. Environ. Res..

[15] Huang L., Zhang X., Wang L., Liu T., Li D., Sheng T., Sun C., Li L. (2025). Boosting catalytic performance of N doped porous carbon derived from coal tar pitch: The role of N species and the contribution of ^1^O_2_. J. Water Process Eng..

[16] Zheng K., Xiao L. (2022). Iron and nitrogen co-doped porous carbon derived from natural cellulose of wood activating peroxymonosulfate for degradation of tetracycline: Role of delignification and mechanisms. Int. J. Biol. Macromol..

[17] Chen F., Zhao R., Zhao H., Liu Q., Zhao S., Li N., Shen Z., Bu X.H. (2025). Axial Co-O-Cu Pairs Enable Superexchange Interaction for Efficient Co^IV^=O Formation toward Water Purification. J. Am. Chem. Soc..

[18] Liu S., Zhang Z., Huang F., Liu Y., Feng L., Jiang J., Zhang L., Qi F., Liu C. (2021). Carbonized polyaniline activated peroxymonosulfate (PMS) for phenol degradation: Role of PMS adsorption and singlet oxygen generation. Appl. Catal. B.

[19] Byambaa B., Seid M.G., Song K.G., Kim E.J., Lee D., Lee C. (2024). Insight into disparate nonradical mechanisms of peroxymonosulfate and peroxydisulfate activation by N-doped oxygen-rich biochar: Unraveling the role of active sites. Chemosphere.

[20] Bao Y., Liu W., Cao J., Zhang J., Xing M. (2023). Self-Neutralized Conditions Constructed by Amphoteric Zinc in Cobalt-Induced Peroxymonosulfate Activation for Sustainable Degradation of Organic Pollutants. ACS ES&T Eng..

[21] Xing P., Wang Y., Fan X., Li X., Wang P., Zhang Q., Du Q., Xie Y., Yin R., Gan W. (2025). Rapid Synthesis and Recycling of Carbonized Wood Catalyst Decorated with Co Nanoparticles for High-Efficiency Degradation of Rhodamine B. Adv. Funct. Mater..

[22] Li X., Min X., Hu X., Jiang Z., Li C., Yang W., Zhao F. (2021). In-situ synthesis of highly dispersed Cu-CuxO nanoparticles on porous carbon for the enhanced persulfate activation for phenol degradation. Sep. Purif. Technol..

[23] Rao R., Huang Y., Zhang H., Hu C., Dong X., Fang W., Zhou Q., Chen Z., Fang S., Jin D. (2024). A simple melamine-assisted cellulose pyrolysis synthesis of magnetic and mesoporous N-doped carbon composites with excellent adsorption of Congo red. Sep. Purif. Technol..

[24] Leng L., Xu S., Liu R., Yu T., Zhuo X., Leng S., Xiong Q., Huang H. (2020). Nitrogen containing functional groups of biochar: An overview. Bioresour. Technol..

[25] Amaral M.S.C., da Silva M.A., Cidade G.d.S., Faria D.N., Cipriano D.F., Freitas J.C.C., dos Santos F.S., Pietre M.K., dos Santos A.M. (2025). Enhanced Ammonium Removal from Wastewater Using FAU-Type and BEA-Type Zeolites and Potential Application on Seedling Growth: Towards Closing the Waste-to-Resource Cycle. Processes.

[26] Zhu Z.S., Wang Y., Duan X., Wang P., Zhong S., Ren S., Xu X., Gao B., Vongsvivut J.P., Wang S. (2024). Atomic-Level Engineered Cobalt Catalysts for Fenton-Like Reactions: Synergy of Single Atom Metal Sites and Nonmetal-Bonded Functionalities. Adv. Mater..

[27] Liu S., Du J., Wang H., Jia W., Wu Y., Qi P., Zhan S., Wu Q., Ma J., Ren N. (2024). How hetero-single-atom dispersion reconstructed electronic structure of carbon materials and regulated Fenton-like oxidation pathways. Water Res..

[28] Tian Y., Xiao J., Pan F., Yu S., Li Z. (2026). One-step activation-graphitization engineering of coal-derived activated carbons with enhanced graphitization for ultrahigh-rate supercapacitors. Chem. Eng. J..

[29] Tang X., Dong T., Wang M., Ma S., Xu S., Wang J., Gao B., Huang Y., Yang Q., Hua D. (2024). From waste corn straw to graphitic porous carbon: A trade-off between specific surface area and graphitization degree for efficient peroxydisulfate activation. J. Hazard. Mater..

[30] Zhang H., Hwang S., Wang M., Feng Z., Karakalos S., Luo L., Qiao Z., Xie X., Wang C., Su D. (2017). Single Atomic Iron Catalysts for Oxygen Reduction in Acidic Media: Particle Size Control and Thermal Activation. J. Am. Chem. Soc..

[31] Wen Q., Wang J. (2025). Degradation of sulfamethoxazole using peroxymonosulfate activated by nitrogen-doped biochar: Insights into the active sites and activation mechanism. Chem. Eng. J..

[32] Li O.L., Prabakar K., Kaneko A., Park H., Ishizaki T. (2019). Exploration of Lewis basicity and oxygen reduction reaction activity in plasma-tailored nitrogen-doped carbon electrocatalysts. Catal. Today.

[33] Zhang W., Li M., Shang W., Wang M., Zhang J., Sun F., Li M., Li X. (2023). Singlet oxygen dominated core-shell Co nanoparticle to synergistically degrade methylene blue through efficient activation of peroxymonosulfate. Sep. Purif. Technol..

[34] Mao Y., Ma W., Zuo W., Sun H., Tian Y., Zhang J., Li L. (2026). Breaking planar confinement: Tailoring the curvature of nanoconfined catalysts to activate PMS for efficient ^1^O_2_ generation. Appl. Catal. B Environ. Energy.

[35] Song J., Hou N., Liu X., Bi G., Wang Y., Mu Y. (2024). Directional Formation of Reactive Oxygen Species Via a Non-Redox Catalysis Strategy That Bypasses Electron Transfer Process. Adv. Mater..

[36] Chen Z., Zhao C., Ma R., Yao Z., Guo Z., Zhao Z., Zheng Y., Fu H. (2025). Interface and Polarization Engineering of ZIF-67(Co)-Based Heterojunctions for Selective Non-Radical PMS Activation: Simultaneous Generation of ^1^O_2_ and Co(IV)=O for Efficient and Safe Water Remediation. Adv. Funct. Mater..

[37] Wang S., Xu L., Wang J. (2019). Nitrogen-doped graphene as peroxymonosulfate activator and electron transfer mediator for the enhanced degradation of sulfamethoxazole. Chem. Eng. J..

[38] Peng C., Wang Q., Zhang X., Dong L., Yuan Y., Zhang M., Rao P., Gao N., Deng J. (2025). Efficient degradation of SCP by Co_2_(OH)_2_CO_3_/CuCo_2_S_4_-enhanced electron transfer-activated PMS: Dual role of Cu active site. Sep. Purif. Technol..

[39] Chai Y., Dai H., Zhan P., Liu Z., Huang Z., Tan C., Hu F., Xu X., Peng X. (2023). Selective degradation of organic micropollutants by activation of peroxymonosulfate by Se@NC: Role of Se doping and nonradical pathway mechanism. J. Hazard. Mater..

[40] Luo M., Zhang H., Zhou P., Xiong Z., Huang B., Peng J., Liu R., Liu W., Lai B. (2022). Efficient activation of ferrate(VI) by colloid manganese dioxide: Comprehensive elucidation of the surface-promoted mechanism. Water Res..

[41] Xing T., Zheng Y., Li L.H., Cowie B.C.C., Gunzelmann D., Qiao S.Z., Huang S., Chen Y. (2014). Observation of Active Sites for Oxygen Reduction Reaction on Nitrogen-Doped Multilayer Graphene. ACS Nano.

[42] Guo D., Shibuya R., Akiba C., Saji S., Kondo T., Nakamura J. (2016). Active sites of nitrogen-doped carbon materials for oxygen reduction reaction clarified using model catalysts. Science.

[43] Ganguly A., Sharma S., Papakonstantinou P., Hamilton J. (2011). Probing the Thermal Deoxygenation of Graphene Oxide Using High-Resolution In Situ X-ray-Based Spectroscopies. J. Phys. Chem. C.

[44] Xiao B., Boudou J.P., Thomas K.M. (2005). Reactions of Nitrogen and Oxygen Surface Groups in Nanoporous Carbons under Inert and Reducing Atmospheres. Langmuir.

[45] Cheng J., Lyu C., Chen H., Geng D., Zheng J. (2024). Strong Lewis acid-base interfacial regulation mechanism to reveal oxygen reduction activity origin of N,S-codoped carbon with PtNi particles. Chem. Eng. J..

[46] Wang T., Chen Z.-X., Chen Y.-G., Yang L.-J., Yang X.-D., Ye J.-Y., Xia H.-P., Zhou Z.-Y., Sun S.-G. (2018). Identifying the Active Site of N-Doped Graphene for Oxygen Reduction by Selective Chemical Modification. ACS Energy Lett..

[47] Ficca V.C.A., Santoro C., D’Epifanio A., Licoccia S., Serov A., Atanassov P., Mecheri B. (2020). Effect of Active Site Poisoning on Iron−Nitrogen−Carbon Platinum-Group-Metal-Free Oxygen Reduction Reaction Catalysts Operating in Neutral Media: A Rotating Disk Electrode Study. ChemElectroChem.

[48] Liu S., Yin S., Zhang Z., Feng L., Liu Y., Zhang L. (2023). Regulation of defects and nitrogen species on carbon nanotube by plasma-etching for peroxymonosulfate activation: Inducing non-radical/radical oxidation of organic contaminants. J. Hazard. Mater..

[49] Yang D., Velamakanni A., Bozoklu G., Park S., Stoller M., Piner R.D., Stankovich S., Jung I., Field D.A., Ventrice C.A. (2009). Chemical analysis of graphene oxide films after heat and chemical treatments by X-ray photoelectron and Micro-Raman spectroscopy. Carbon.

[50] Diallo-Garcia S., Osman M.B., Krafft J.-M., Casale S., Thomas C., Kubo J., Costentin G. (2014). Identification of Surface Basic Sites and Acid–Base Pairs of Hydroxyapatite. J. Phys. Chem. C.

[51] Wang X., Lu L., Wang B., Xu Z., Xin Z., Yan S., Geng Z., Zou Z. (2018). Frustrated Lewis Pairs Accelerating CO_2_ Reduction on Oxyhydroxide Photocatalysts with Surface Lattice Hydroxyls as a Solid-State Proton Donor. Adv. Funct. Mater..

[52] Peng Y., Xiao W., Wang H., Bian Z. (2025). Carbon defect induced electron transfer promotes electrocatalytic activation of molecular oxygen to selectively generate singlet oxygen for pollutants removal. Appl. Catal. B Environ. Energy.

[53] Zhao Z., Yang G., Wang P., Yue S., Yang M., Zhang T., Zhan S. (2025). Regulating nonradicals generation through peroxymonosulfate activation via localized dipole to enhance wastewater biodegradability. Nat. Commun..

[54] Wang X., Wang W., Peng J., Wei P., Yan G., Cao Z. (2025). Boosting effect of oxygen vacancy on peroxymonosulfate activation over CoWO_4_ for sulfapyridine removal. Sep. Purif. Technol..

[55] Wang S., Wang K., Cao W., Qiao L., Peng X., Yu D., Wang S., Li C., Wang C. (2023). Degradation of methylene blue by ellipsoidal β-FeOOH@MnO_2_ core-shell catalyst: Performance and mechanism. Appl. Surf. Sci..

[56] Zhang H., Zhou C., Zeng H., Shi Z., Wu H., Deng L. (2022). Novel sulfur vacancies featured MIL-88A(Fe)@CuS rods activated peroxymonosulfate for coumarin degradation: Different reactive oxygen species generation routes under acidic and alkaline pH. Process Saf. Environ. Prot..

[57] Liu X., Lu L., Zhu M., Englert U. (2021). Design and synthesis of three new copper coordination polymers: Efficient degradation of an organic dye at alkaline pH. Dalton Trans..

