# Supplementary material for: Deactivation and Regeneration of Lewis Basic Sites Following Reversible Chemical Adsorption and Desorption of Hydroxyl Groups in Contaminant Degradation by Advanced Oxidation
Source: Materials (Basel). 2026 Apr 15;19(8):1589. doi: 10.3390/ma19081589 (PMC13117196; doi:10.3390/ma19081589)
Supplement: Supplementary file 1 [file materials-19-01589-s001.zip › materials-4236674-supplementary.pdf]

Supplementary Information

Deactivation and regeneration of Lewis-basic sites following reversibly chemical adsorption and desorption of hydroxyl groups in contaminants degradation by advanced oxidation

Lekang Zhao, Huailin Fan [*], *Juncheng Zhao, Xixi Zhang, Xiaohang Ma, Xun Hu*[**], *Qingyu Ma* [***]

School of Material Science and Engineering, University of Jinan, Jinan, 250022, P. R. China.

*Corresponding author. Email: huailinfan@163.com

**Corresponding author. Email: xun.hu@outlook.com

***Corresponding author. Email: mse_maqy@ujn.edu.cn

Text S1

Reagents

The heavy oil came from a biomass processing company in Nanjing, China. The reagents provided by Shanghai Macklin Biochemical Co., Ltd. Included melamine, Magnesium citrate(9-hydrat), furfuryl alcohol (FFA), SMZ, RhB, MCPA, BPA, PMS ($KHSO_5 \cdot 0.5KHSO_4 \cdot 0.5K_2SO_4$). 5,5-dimethyl-1-pyrroline-N-oxide (DMPO), and 2,2,6,6-tetramethylpiperidine (TEMP) were supplied by Aladdin Inc. Methanol (MeOH), ethanol (EtOH), sodium chloride (NaCl), furfuralcohol (FFA), sodium hydroxide (NaOH), and hydrochloric acid (HCl) were all obtained from Beijing Chemical Works. Sodium carbonate (Na_2CO_3), sodium sulfate (Na_2SO_4), and potassium nitrate (KNO_3) were supported by Xilong Scientific Co., Ltd.

Text S2

Characterization of catalysts

Transmission electron microscopy (HRTEM) images was collected on a JEM-2100 Plus microscope (JEOL, Japan) at an accelerating voltage of 200 kV. X-ray photoelectron spectroscopy (XPS) was operated on a photoelectron spectrometer (Escalab 250Xi, Thermo Fisher, USA) with Al Ka x-ray source. Raman spectra were obtained through a Raman spectrometer (LabRAM HR Evolution, Horiba, France). Elemental analyzer (EA, EuroEA3000-Single, Italy) was employed to assess C, H, and N concentrations, with oxygen content estimated by calculating the difference. Fourier transform infrared spectroscopy (FTIR, Thermo Fisher Nicolet iS50, USA) was utilized to investigate the distribution of surface functional groups. The microstructure of solid materials was studied using scanning electron microscopy (SEM, EM-30, Korea). Nitrogen adsorption-desorption isotherms (BET, BIAODE SSA-6000, China) were employed. The crystal structure was determined via X-ray diffraction (XRD, Rigaku Ultima IV) using Cu Kα radiation, with samples scanned from 5° to 80° at 20°/min. The step size was set to 0.02°. During degradation experiments, solute concentrations were measured using ultraviolet-visible spectrophotometry (T9S, China).

Text S3

Electrochemical experiments

Electrochemical experiments were carried out with a three-electrode-cell system containing a working electrode (glassy carbon), a reference electrode (Ag/AgCl), and counter electrode (Pt). For the preparation of the NC-900-coated glassy carbon electrode (NC-900/GCE), 5 mg NC-900 and 50 uL Nafion were added to 1 mL ethanol solution, followed by sonication for 30 min. Then, the resulting suspension (5 μL) was deposited onto the glassy carbon electrode and allowed to dry at room temperature before use. The electrolyte was Na_2SO_4 solution (20 mM). When measuring the open circuit potential (OCP), the prepared NC-900/GCE was first immersed in 100 mL Na_2SO_4 solution to stabilize the potential. 3 mL PMS (0.1 M) was added to Na_2SO_4 solution at 400 s and observed the voltage change. Then, 2.5 mL MB solution (0.2 g L−1) was added at 800 s and continued observing the voltage change until 1200 s. In addition to Na_2SO_4 solution, two other solutions were prepared for linear sweep voltammetry (LSV) testing: one was a Na_2SO_4 solution containing only PMS (3 mM), and the other was a Na_2SO_4 solution containing both PMS (3 mM) and MB (20 mg L−1). LSV was performed at a potential of 0 V to 1.1 V with a scan rate of 20 mV s−1. Galvanically coupled oxidation system (GOS) was implemented to analyze electron transfer between MB and NC-900. The experimental configuration isolated PMS and MB in dual electrolytic cells connected via an agar salt bridge and ammeter, with NC-900-coated electrodes monitoring real-time current and degradation efficiency during the reaction. Under these conditions, MB oxidation occurred exclusively through electron transfer pathways.

Text S4

Catalysts preparation

The nitrogen-doped catalyst was synthesized with heavy oil and melamine serving as the carbon and nitrogen precursors, respectively. Wherein heavy oil and absolute ethanol were mixed at a mass ratio of 1:10 and magnetically stirred for 30 min. Subsequently, melamine was gradually added to the mixture, and the solution was stirred for an additional 60 min. The mixed solution was dried at 60 °C in a water bath to remove ethanol. The resulting solid was transferred to a graphite crucible, heated to 900 °C at 5 °C min^{-1} under N_2 flow in a tubular furnace, and maintained at this temperature for 2 h. After cooling to room temperature, the resulting product was designated as NC-900. Samples synthesized at 800 °C and 1000 °C were labeled NC-800 and NC-1000, respectively, whereas the control catalyst prepared without melamine was denoted as C-900.

Text S5

Reaction condition:

The MB degradation experiments were carried out in a conical flask containing 50 mL of MB solution (50 mg/L), 3 mM PMS, and 15 mg of catalyst at room temperature (initial pH = 7). The reaction mixture was magnetically stirred at 450 rpm throughout the process. Following an initial sampling at 3 min, subsequent aliquots were withdrawn at 5-min intervals over 30 min. Each aliquot was immediately quenched with methanol to scavenge residual radicals, filtered through a 0.45 μm membrane, and analyzed via UV-Vis spectrophotometry to determine the degradation efficiency and reaction kinetics. The reactive species involved in the degradation process were identified through radical scavenging experiments using furfuryl alcohol (FFA), tert-butanol (TBA), p-benzoquinone (BQ), and ethanol (EtOH). Additionally, the effects of key reaction parameters on catalytic performance were systematically investigated, while the MB degradation kinetics were determined based on pseudo-first-order model fitting. For cycling experiments, catalysts were recovered, washed three times with ethanol followed by water, and dried under vacuum at 60°C for 48 h. All experiments were performed in triplicate to ensure reproducibility.

The intermediates of methylene blue degradation were analyzed by gas chromatography-mass spectrometry (GC-MS) using a C18 column, with conditions including a column oven temperature of 40°C and an injector temperature of 250°C. The temperature program was initially held at 40°C for 3 min, then ramped to 240°C at 20°C/min, and maintained for 8 min.

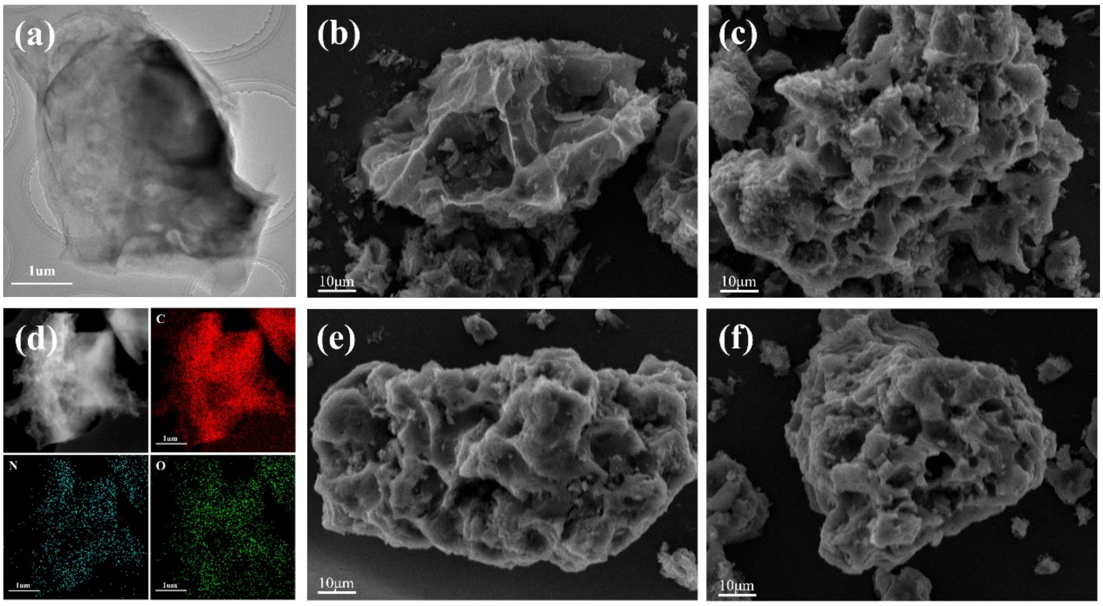

Figure S1. (a) TEM images of NC-900, (b) SEM images of C-900, (c) SEM images of NC-800, (d) Elemental mapping images of NC-900, (e) SEM images of NC-900, (f) SEM images of NC-1000.

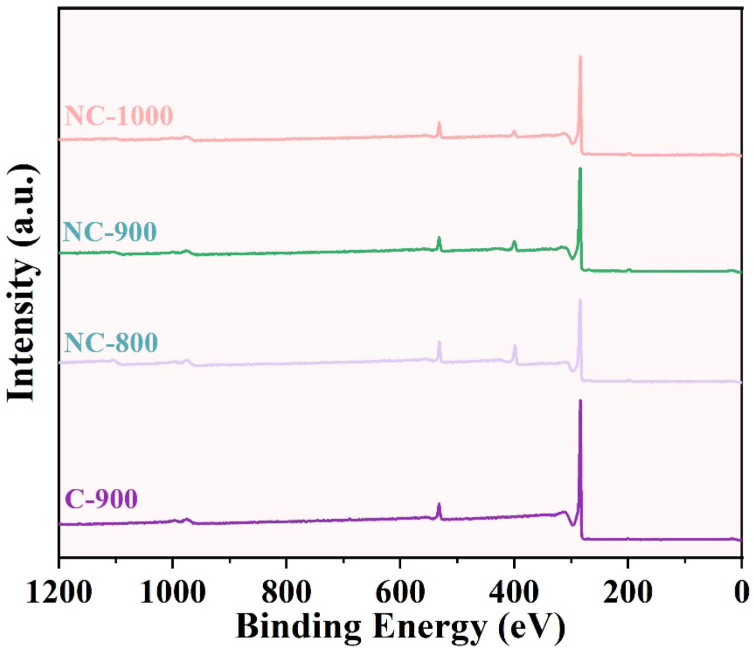

Figure S2. The XPS full-spectrum spectra of C-900, NC-800, NC-900 and NC-100.

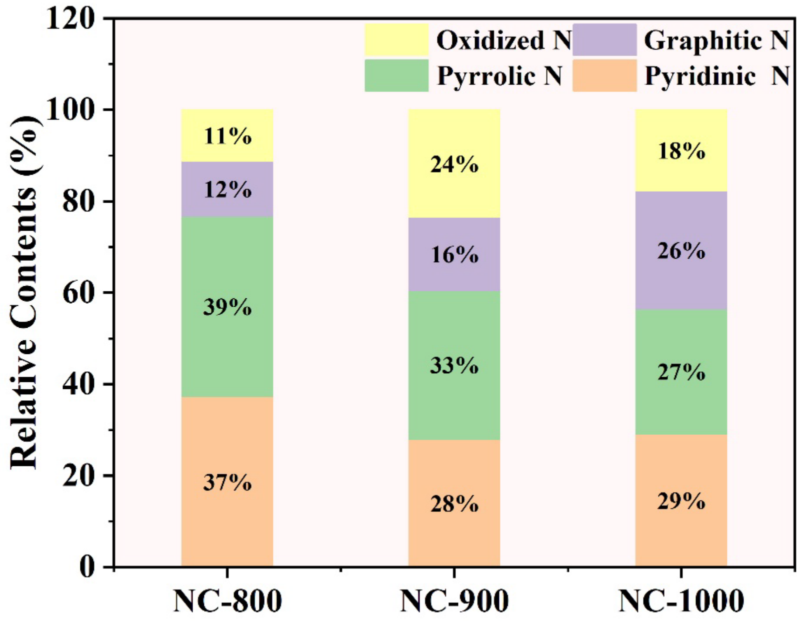

Figure S3. The relative contents of nitrogen species in the XPS spectra of N 1s.

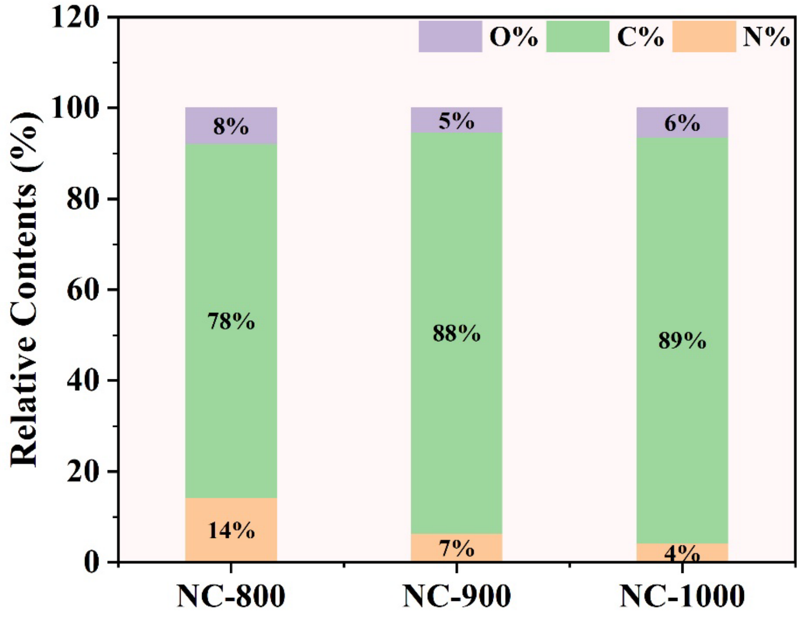

Figure S4. The elemental content in the XPS full-spectrum graph.

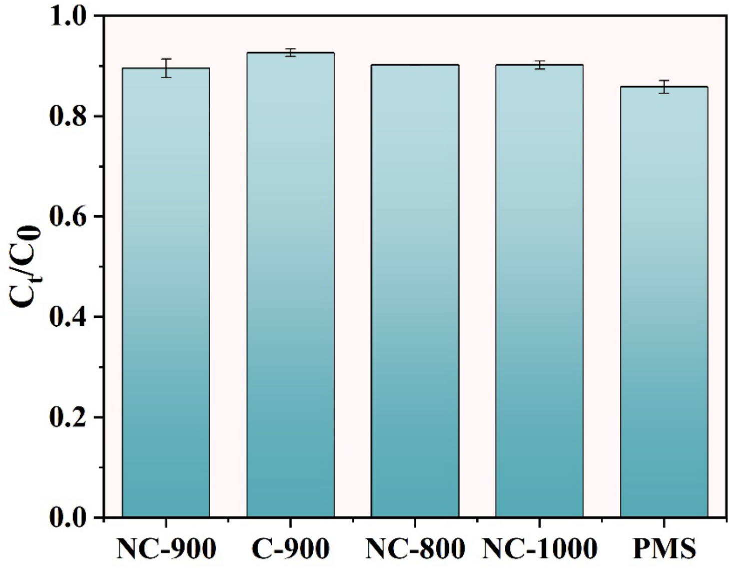

Figure S5. The removal rate of the adsorption effect of the catalysts C-900, NC-800, NC-900 and NC-100 on the MB solution.

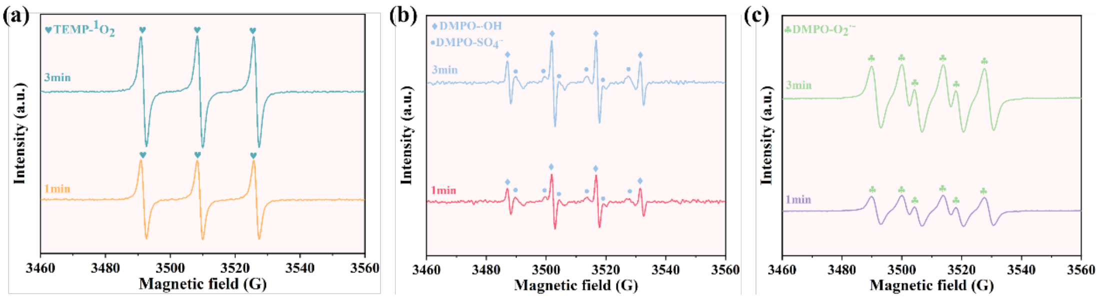

Figure S6. Radical products of catalyst in PMS-assisted ROS production. (a) The temporal variation of TEMP-trapping $^1O_2$ via in situ EPR. (b) The temporal variation of DMPO-trapping •OH and $SO_4^{\bullet -}$ via in situ EPR. (c) The temporal variation of DMPO-trapping $O_2^{\bullet -}$ via in situ EPR.

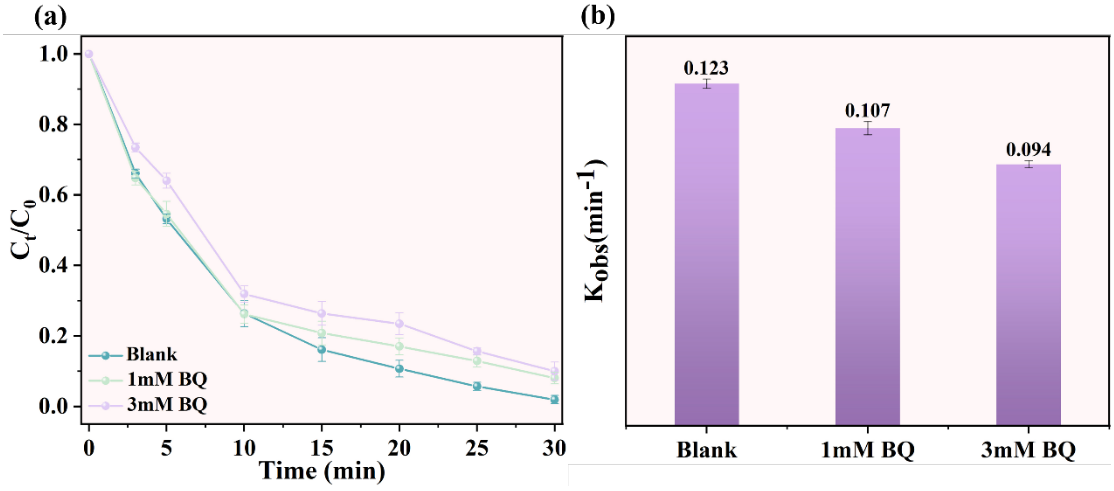

Figure S7. (a) In the NC-900/PMS system, effects of $O_2^{\bullet -}$ radical scavengers on MB degradation (1mMBQ and 3mMBQ).and (b) first-order kinetic curves. Reaction conditions: [catalysts] = 15 mg, [PMS] = 3 mM, [MB] = 50 mg/L, T= 25 °C.

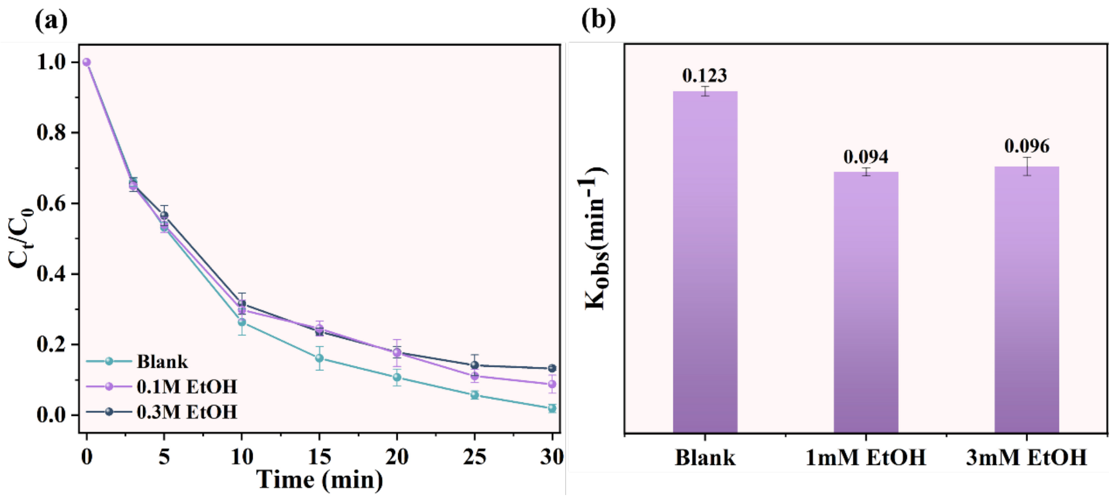

Figure S8. (a)In the NC-900/PMS system, effects of •OH and $SO_4^{\bullet-}$radical scavengers on MB degradation (1mMBQ and 3mMBQ).and (b) first-order kinetic curves. Reaction conditions: [catalysts] = 15 mg, [PMS] = 3 mM, [MB] = 50 mg/L, T= 25 °C.

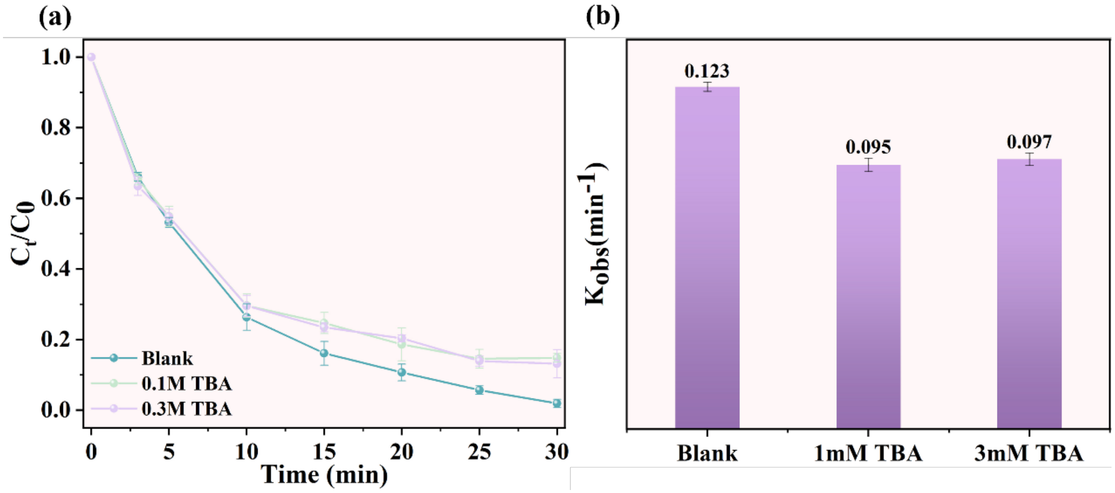

Figure S9. (a) In the NC-900/PMS system, effects of •OH radical scavengers on MB degradation (0.1MTBA and 0.3MTBA).and (b) first-order kinetic curves. Reaction conditions: [catalysts] = 15 mg, [PMS] = 3 mM, [MB] = 50 mg/L, T= 25 °C.

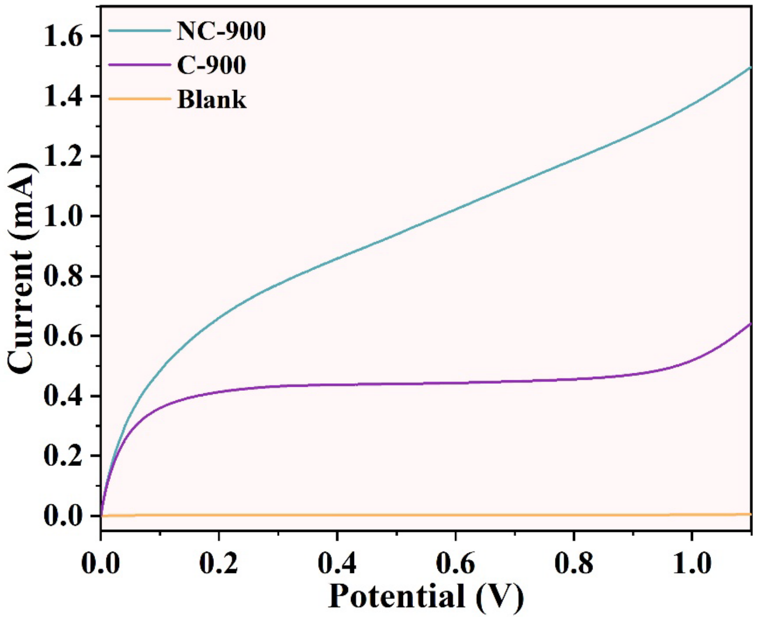

Figure S10. The LSV curve in the C-900、NC-900/PMS system.

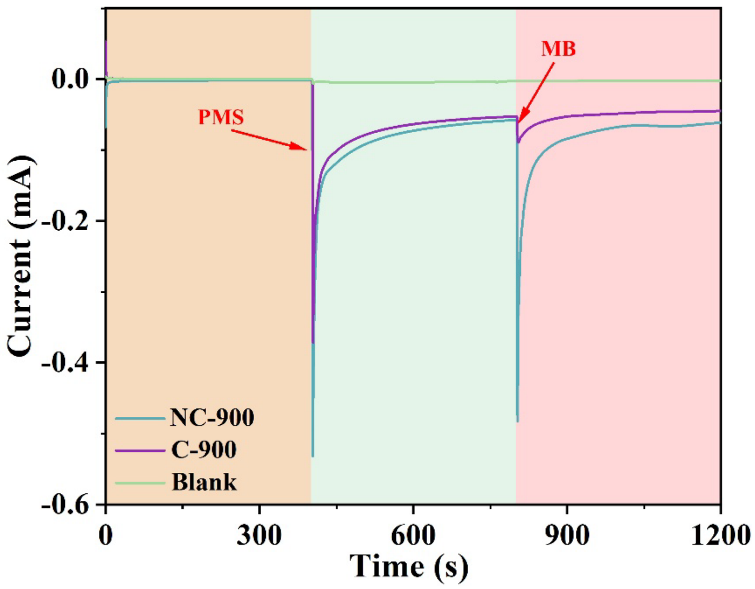

Figure S11. I-t curves in the C-900, NC-900/PMS systems changes with the addition of PMS and MB respectively.

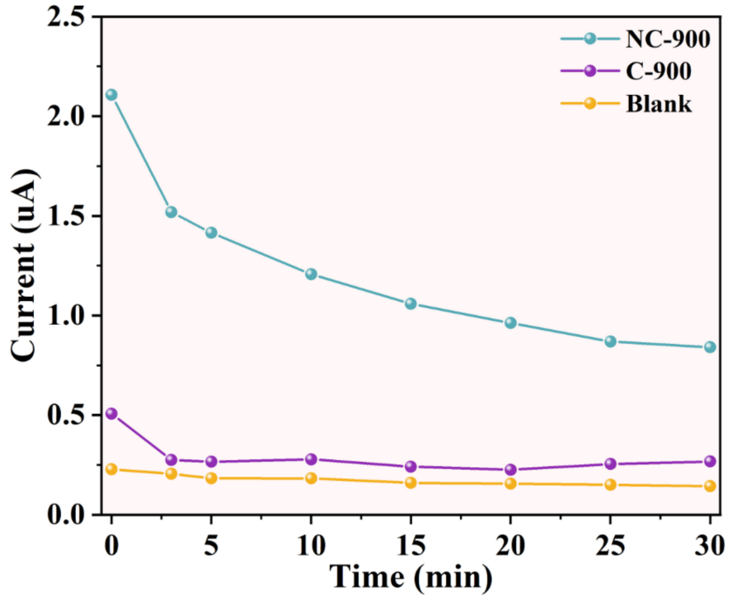

Figure S12. GOS curves for different catalysts. Place PMS and MB solution separately in a dual electrolytic cell connected by an agar salt bridge and an ammeter to achieve complete physical isolation of the two substances. Simultaneously, the working electrode was coated with NC-900, and changes in the system current were monitored.

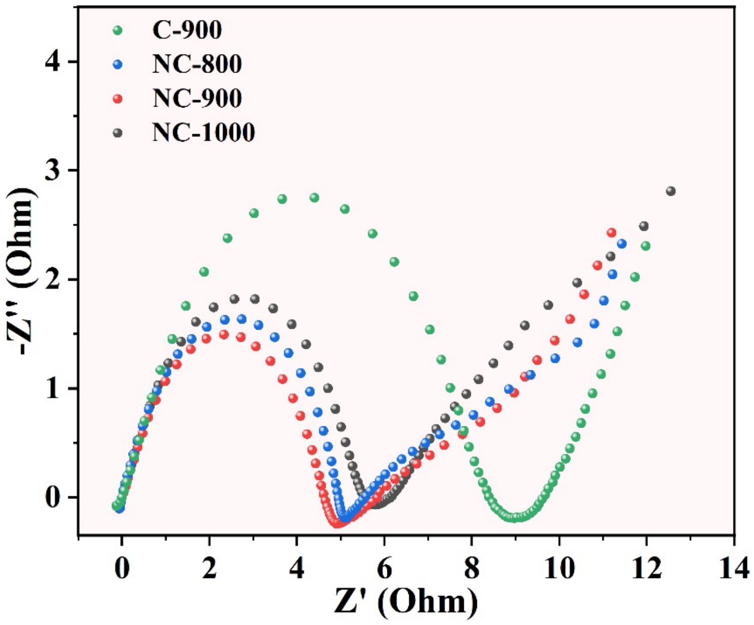

Figure S13. Electrochemical Impedance Spectroscopy of C-900, NC-800, NC-900 and NC-1000.

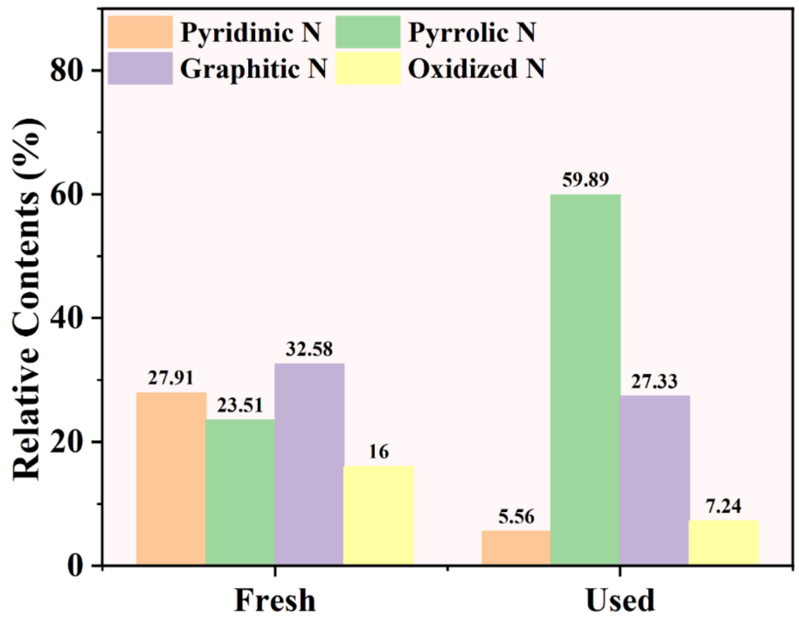

Figure S14. The changes in different nitrogen species in the XPS spectrum of N 1s after NC-900 recovery.

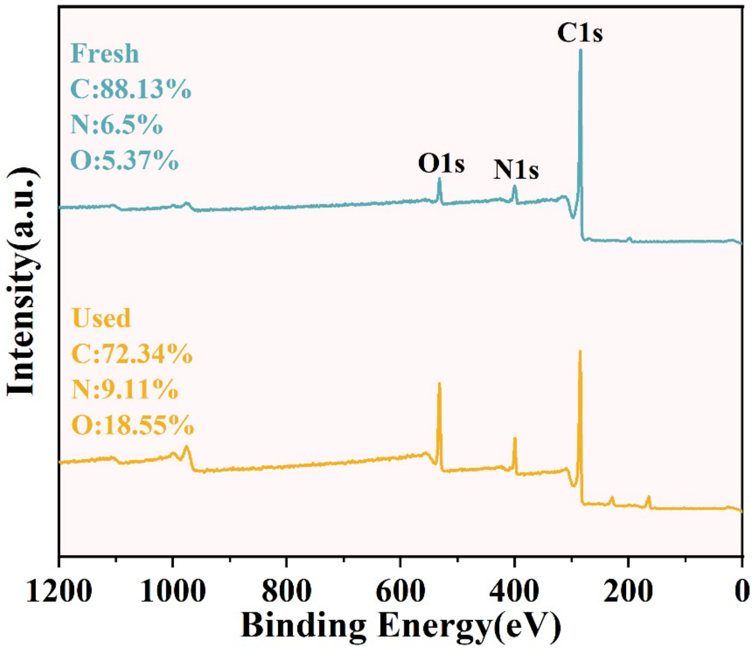

Figure S15. Analysis of the changes in elemental content in the XPS survey spectrum after the recycling of NC-900.

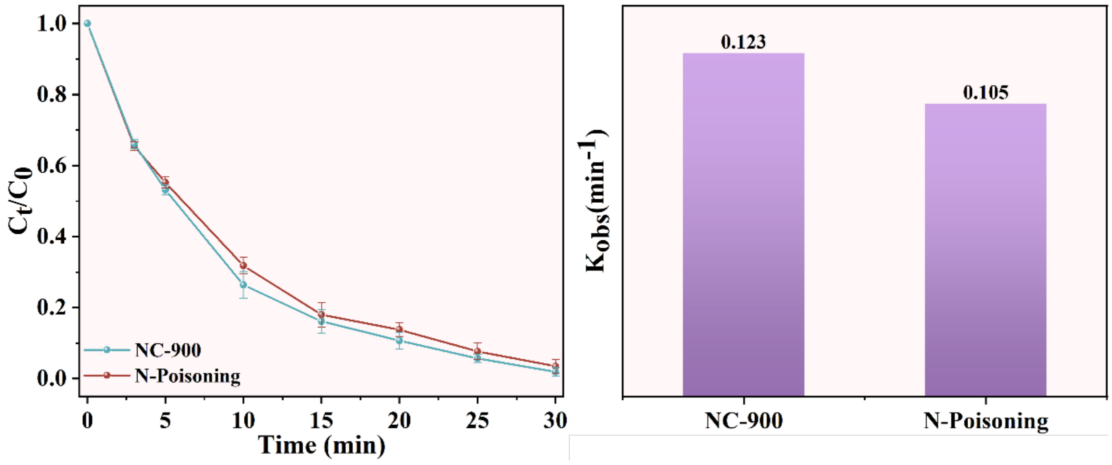

Figure S16. In the NC-900/PMS system, effects of poisoning the nitrogen atom of pyridine on MB degradation. and (b) first-order kinetic curves. Reaction conditions: [catalysts] = 15 mg, [PMS] = 3 mM, [MB] = 50 mg/L, T= 25 °C.

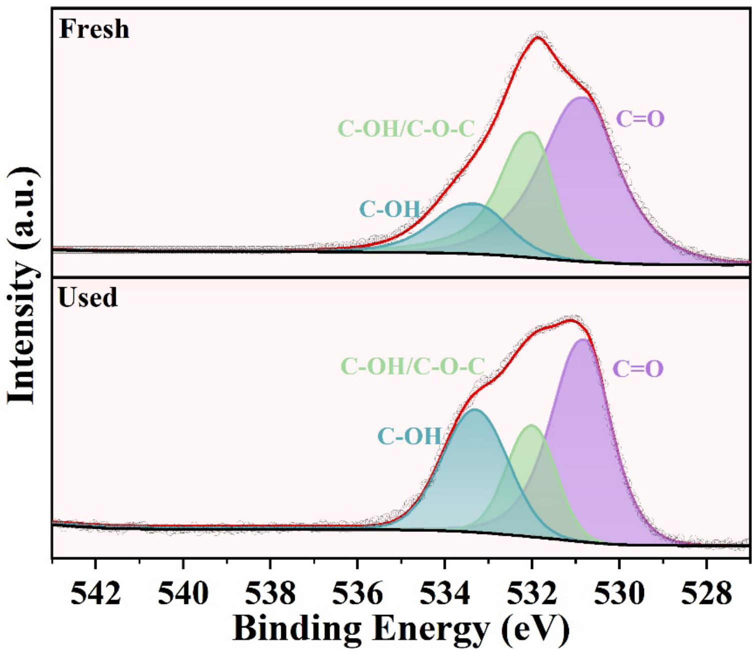

Figure S17. Changes in the oxygen species in the XPS spectra of O 1s after the recycling of NC-900.

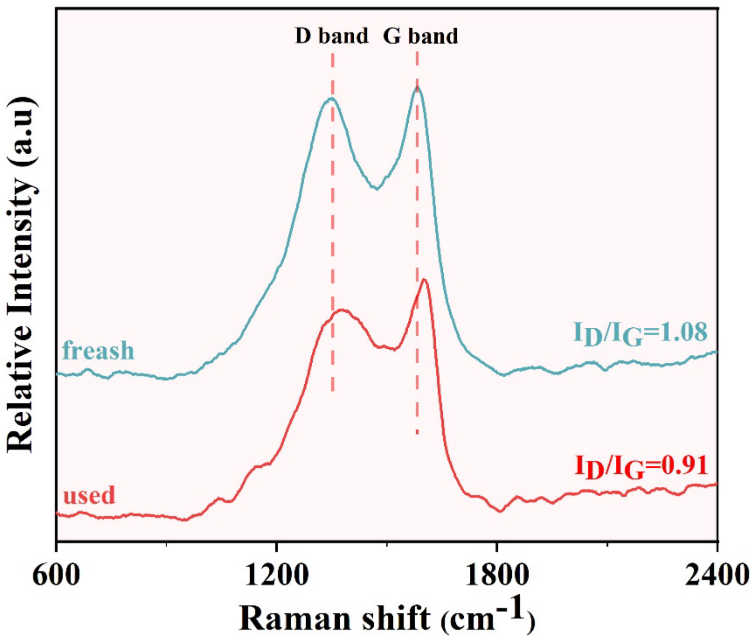

Figure S18. The change in the Raman spectral I_D/I_G ratio after the recycling of NC-900.

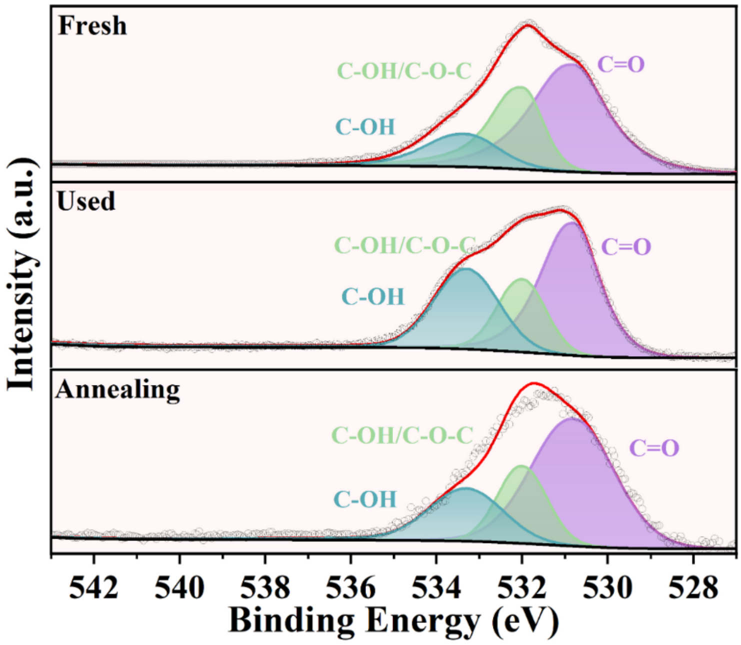

Figure S19. Changes in the oxygen species in the XPS spectra of O 1s after the recycling and thermal regeneration of NC-900.

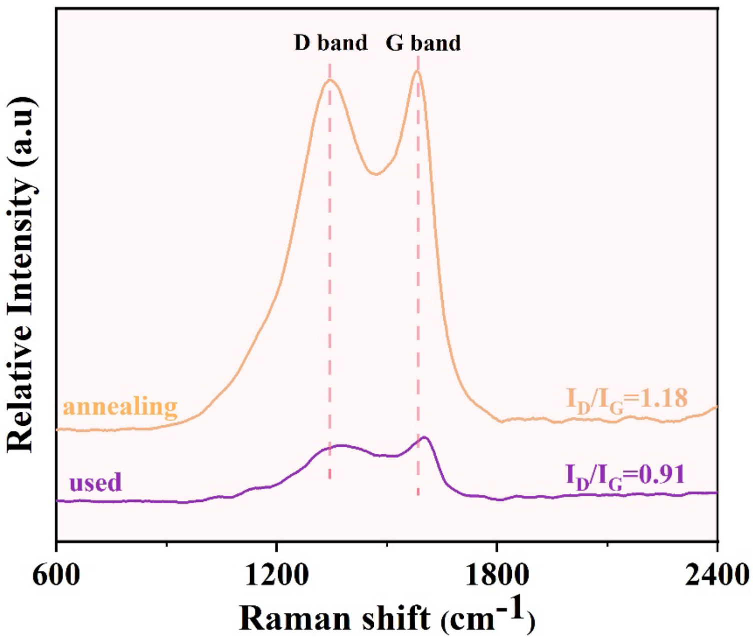

Figure S20. The change in the Raman spectral I_D/I_G ratio after the thermal regeneration of NC-900.

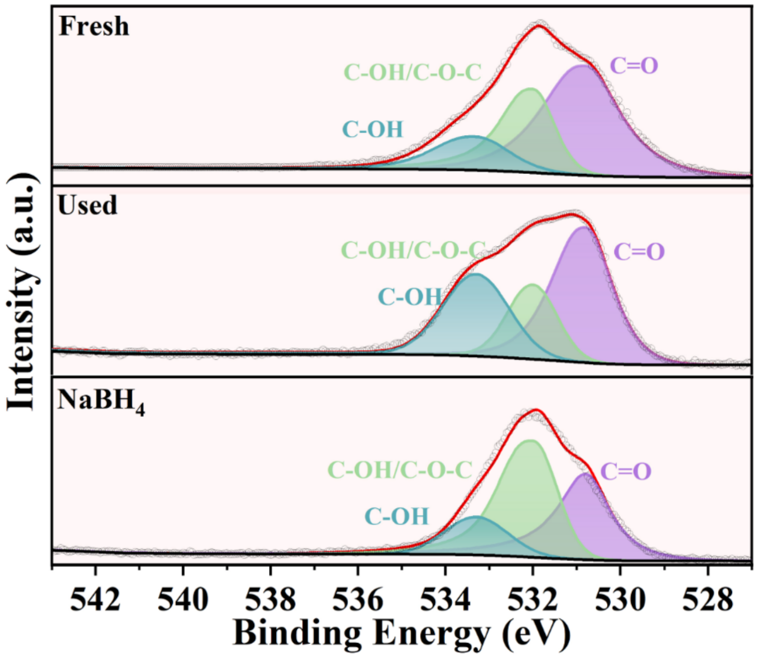

Figure S21. Changes in the oxygen species in the XPS spectra of O 1s after the recycling and $NaBH_4$ regeneration of NC-900.

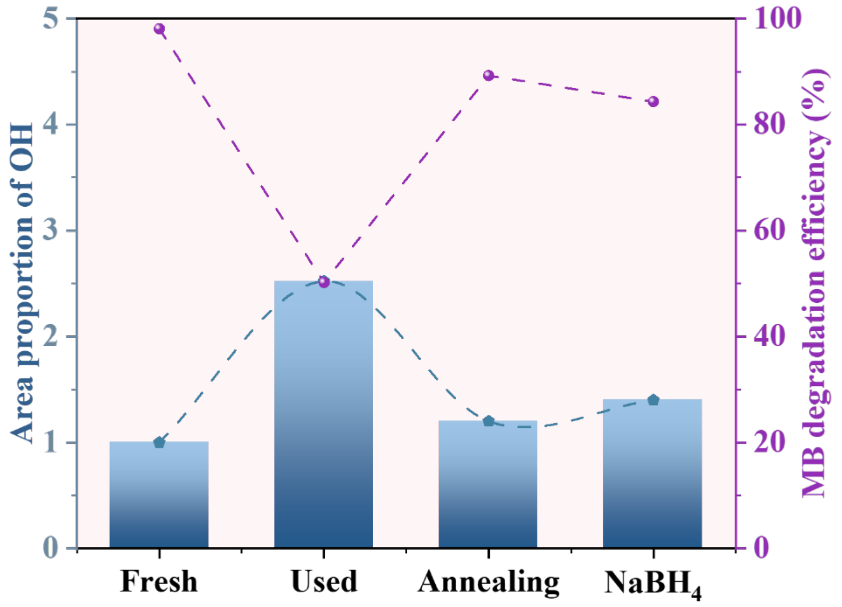

Figure S22. Comparison diagram of the peak area proportion of the OH and Catalytic Efficiency during the catalyst deactivation and regeneration process in the NC-900/PMS degradation MB system.

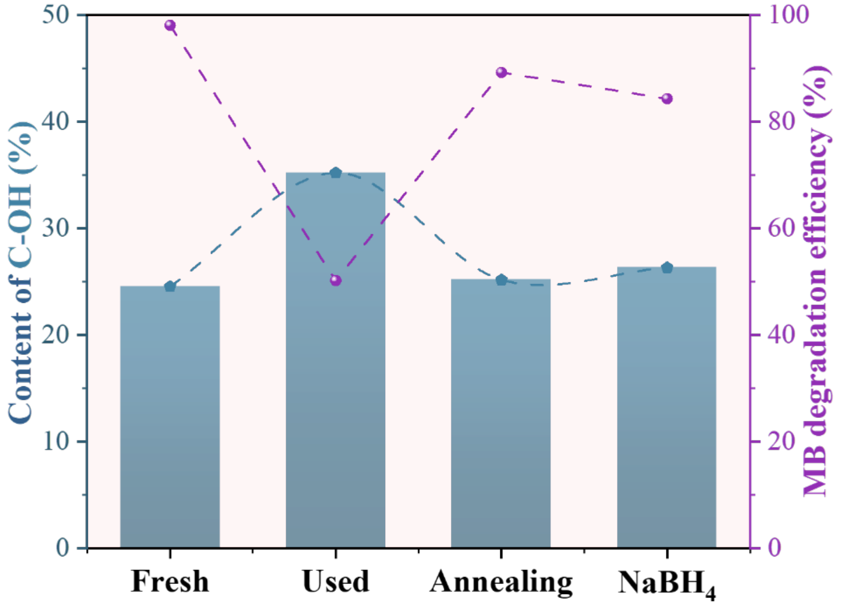

Figure S23. Comparison diagram of Content of C-OH and Catalytic Efficiency during the catalyst deactivation and regeneration process in the NC-900/PMS degradation MB system.

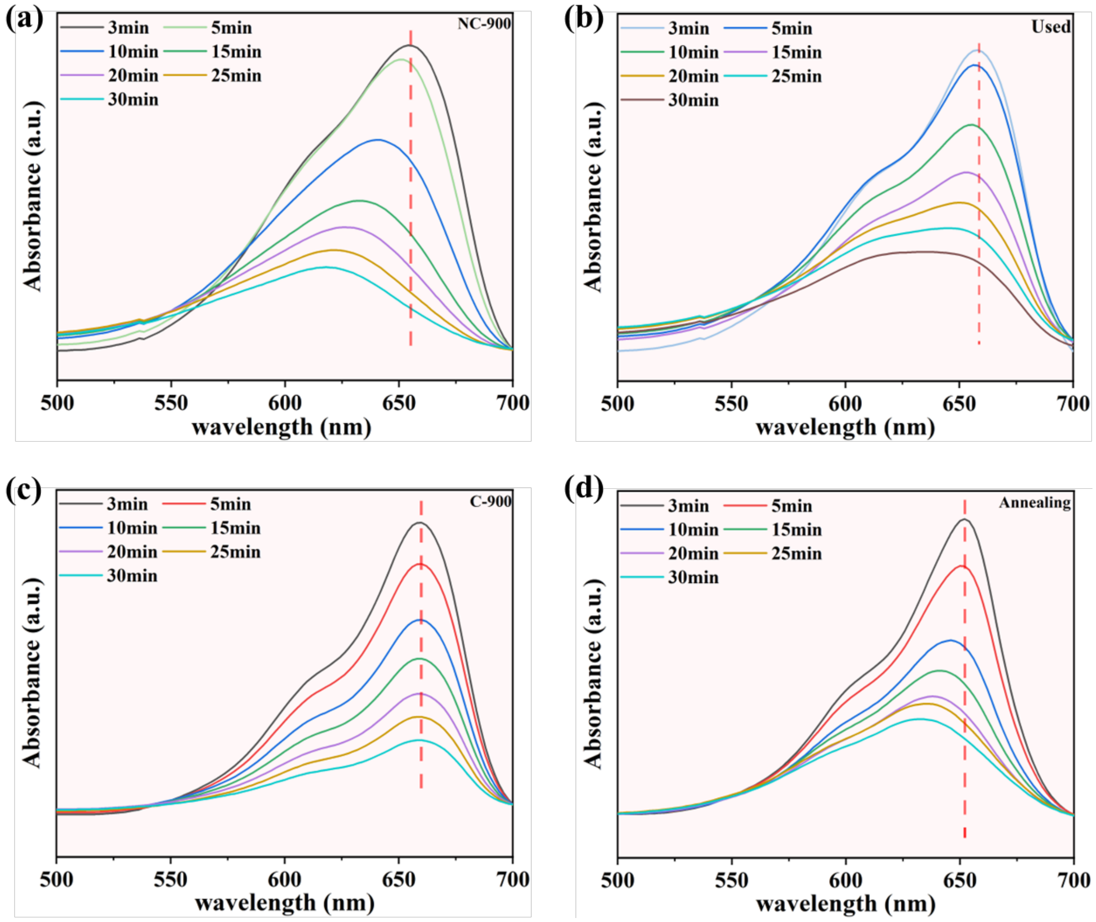

Figure S24. The variation relationship of the ultraviolet concentration curve of MB in different catalyst/PMS systems over time. (a) FreshNC-900, (b) Used NC-900, (c) Fresh C-900, (d) Annealing NC-9000.

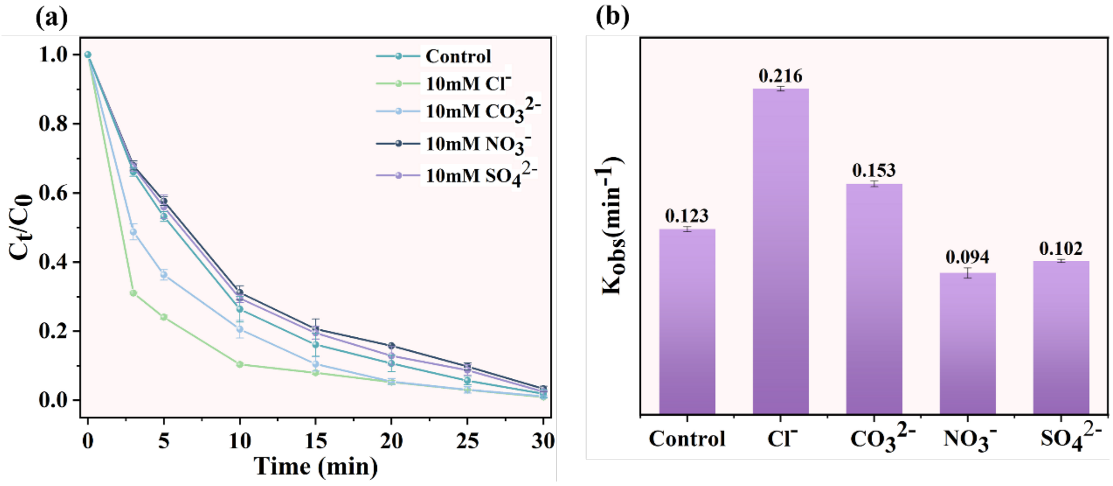

Figure S25. (a) In the NC-900/PMS system, effects of inorganic ion on MB degradation. and (b) first-order kinetic curves. (add 10mMCl^-, 10mMCO_3^{2-},10mMNO_3^-, 10mMSO_4^{2-} respectively) Reaction conditions: [catalysts] = 15 mg, [PMS] = 3 mM, [MB] = 50 mg/L, T= 25 °C.

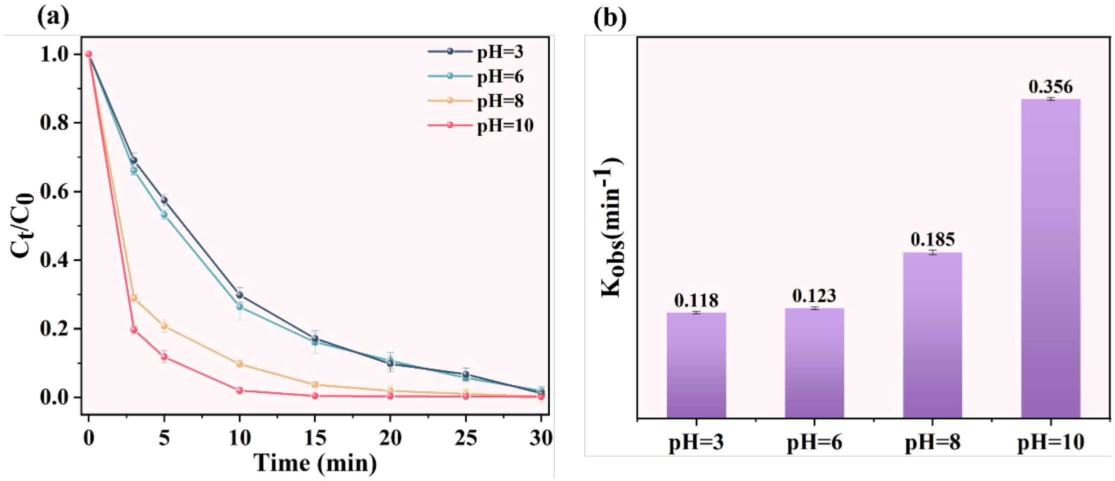

Figure S26. (a) In the NC-900/PMS system, effects of pH on MB degradation. and (b) first-order kinetic curves (pH=3, 6, 8, 10). Reaction conditions: [catalysts] = 15 mg, [PMS] = 3 mM, [MB] = 50 mg/L, T= 25 °C.

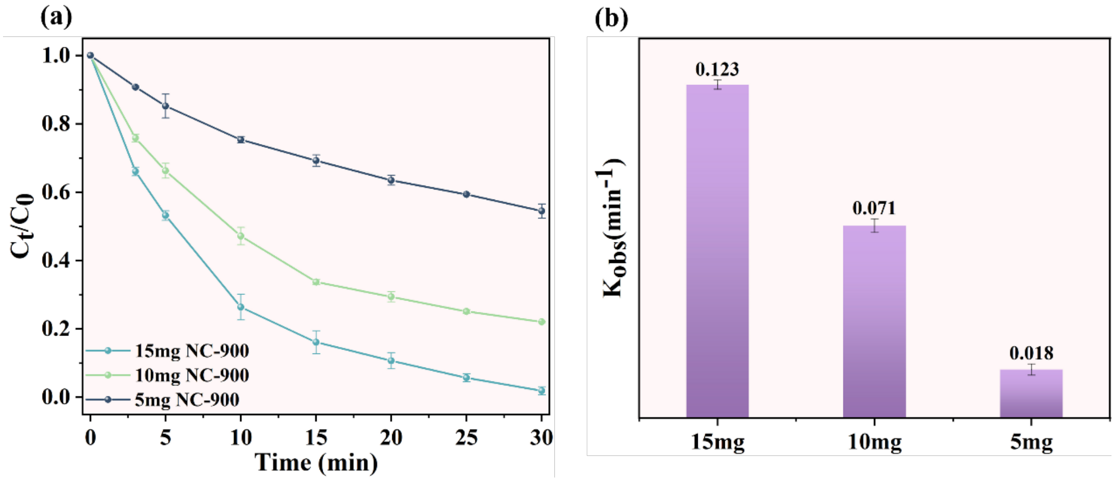

Figure S27. (a) In the NC-900/PMS system, effects of catalyst dosage on MB degradation. and (b) first-order kinetic curves (add 5 mg, 10 mg, 15 mg NC-900 respectively). Reaction conditions: [PMS] = 3 mM, [MB] = 50 mg/L, T= 25 °C.

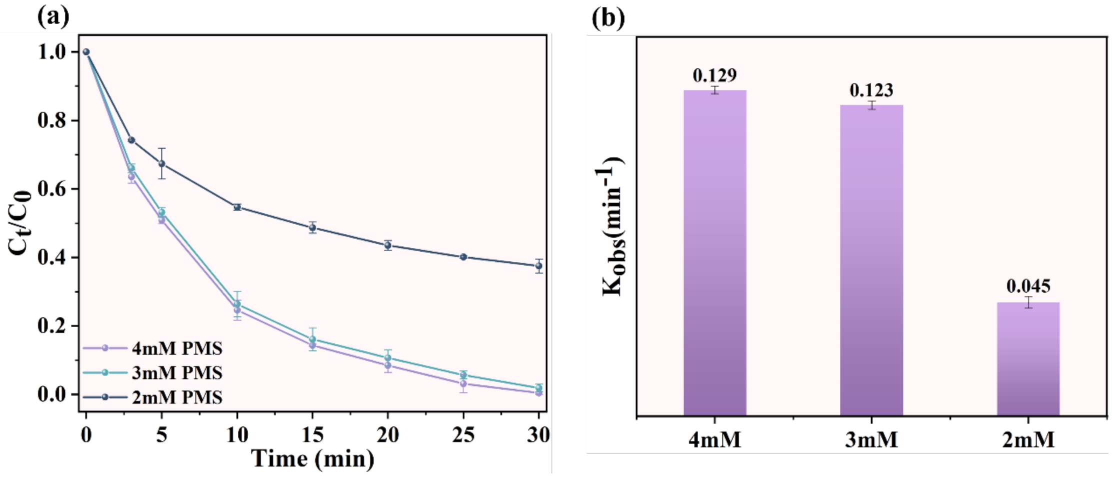

Figure S28. (a) In the NC-900/PMS system, effects of PMS dosage on MB degradation. and (b) first-order kinetic curves (add 2 mM, 3 mM, 4 mM PMS respectively). Reaction conditions: [catalysts] = 15 mg, [MB] = 50 mg/L, T= 25 °C.

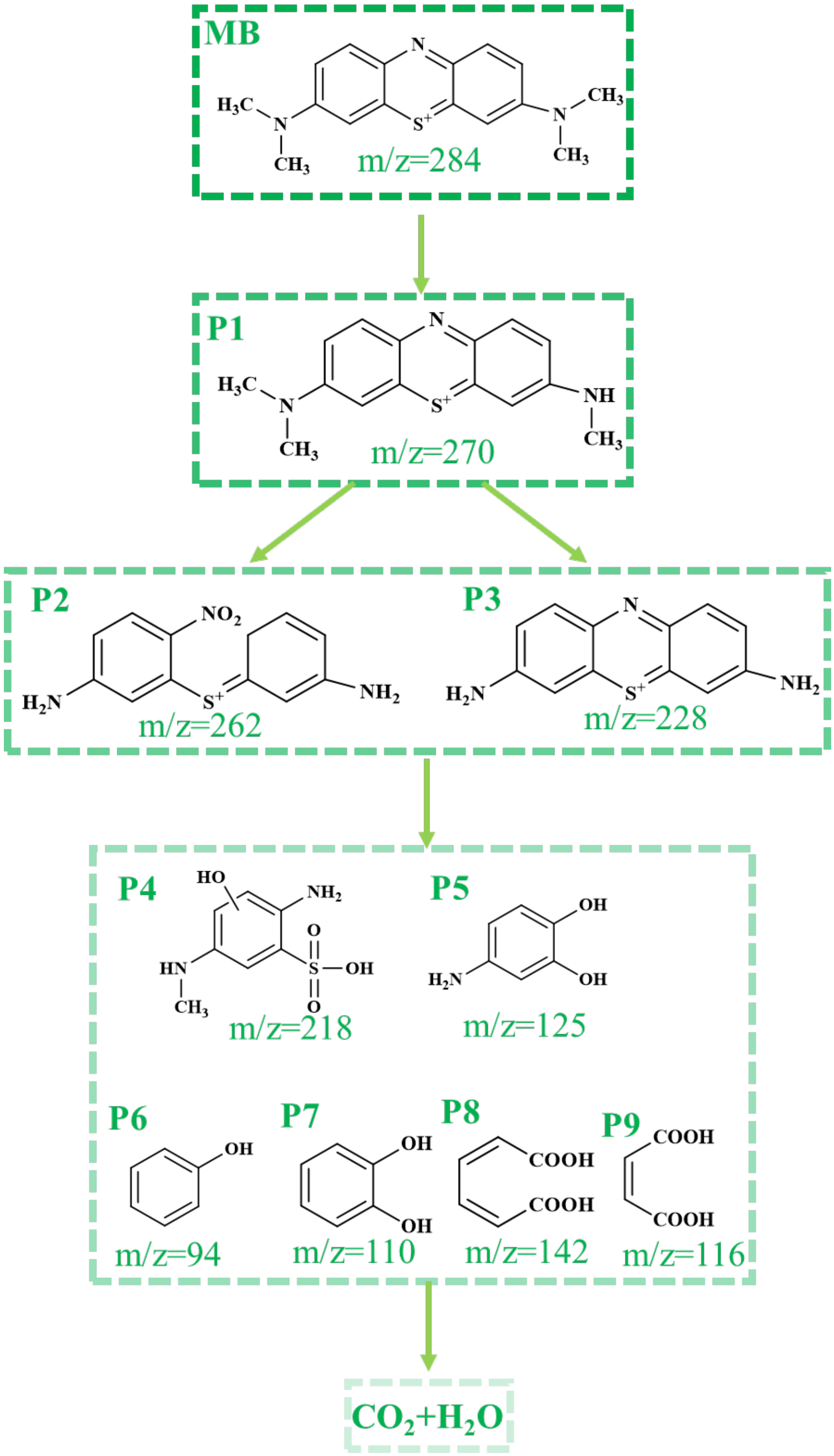

Figure S29. Proposed degradation pathways of MB in NC-900/PMS system.

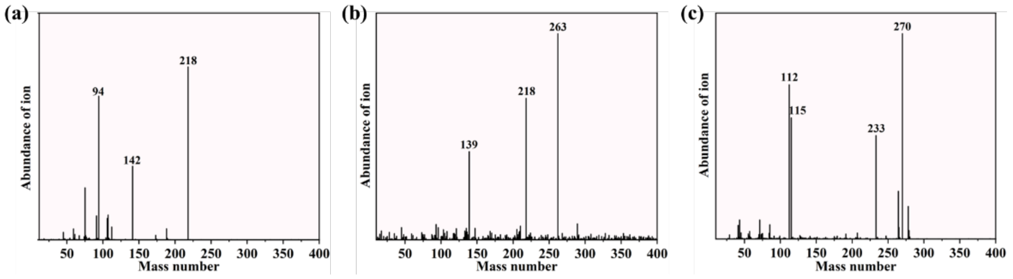

Figure S30. Mass spectrometric analysis of intermediates produced in NC-900/PMS degradation process of MB.

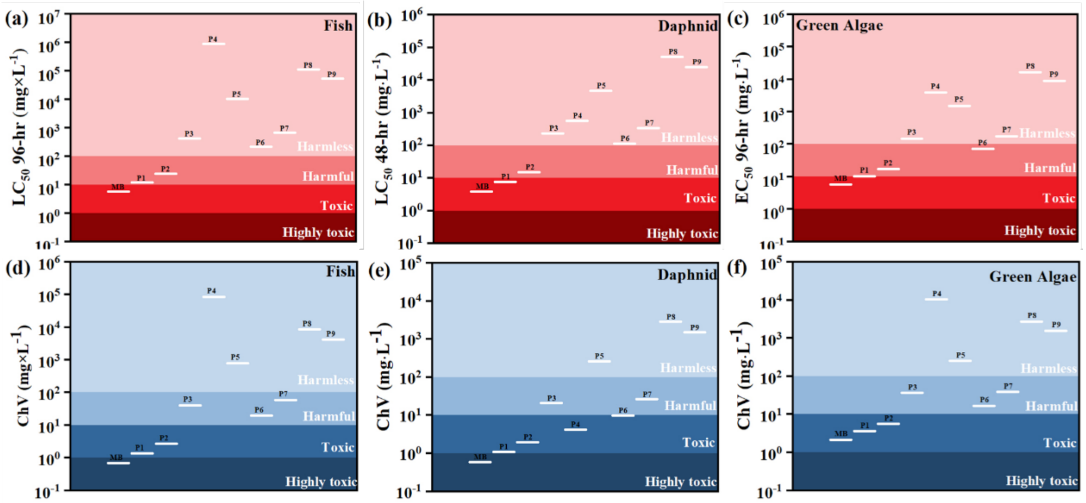

Figure S31. An estimation of the ecotoxicity of MB and its intermediate transformation products was conducted using ECOSAR (a–c: acute toxicity, d–f: chronic toxicity).

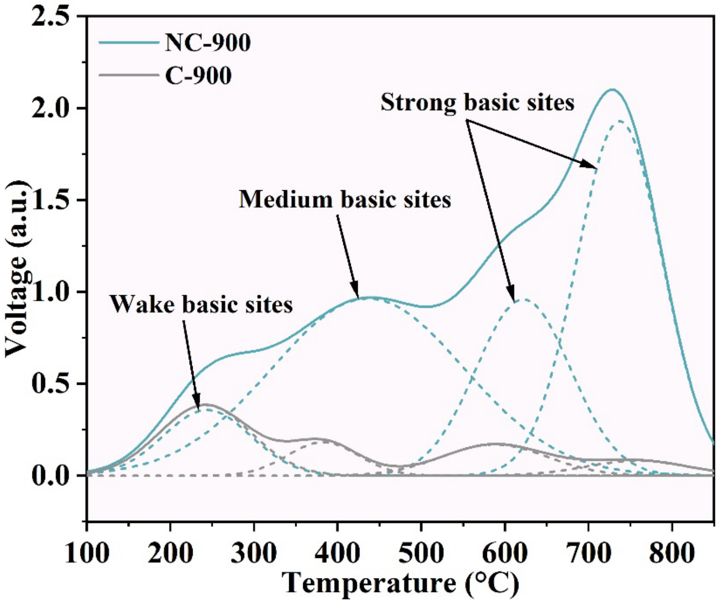

Figure S32. The peak-fitting diagram of the CO_2-TPD curves for the NC-900 and C-900 catalysts

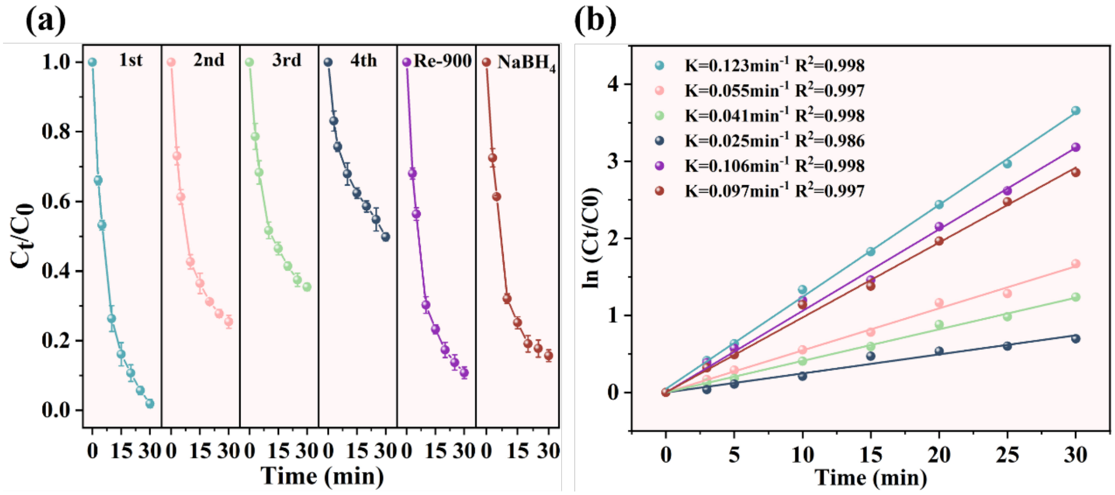

Figure 33. (a)The degradation efficiency of MB after 4 cycles and after regeneration for the NC-900 catalyst. (b) And corresponding pseudo-first-order reaction kinetics.

The cycling stability of the NC-900 catalyst in the NC-900/PMS system for MB degradation was tested, and it was observed that the catalytic efficiency of NC-900 decreased significantly after four cycles, with the corresponding pseudo-first-order kinetic constant declining from 0.123 min^{-1} to 0.025 min^{-1}. However, after thermal regeneration and sodium borohydride reduction regeneration, the catalytic efficiency was markedly enhanced, and the pseudo-first-order kinetic constants increased to 0.106 min^{-1} and 0.197 min^{-1}, respectively. This result demonstrated that both annealing and sodium borohydride reduction treatments effectively removed intermediates attached during the reaction and restored the catalytic activity of the catalyst.

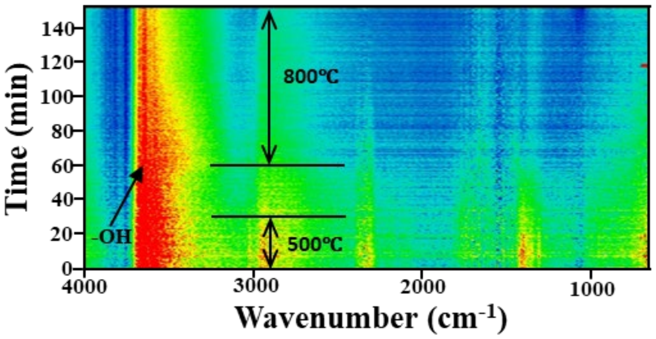

Figure 34. In-situ DRIFTS analysis during the activity regeneration process of NC-900 catalyst

To intuitively demonstrate the reversible deactivation of the Lewis-basic sites induced by hydroxyl group adsorption and the subsequent regeneration of activity upon desorption, the thermal regeneration process of the NC-900 catalyst was monitored by in situ IR spectroscopy, with the results displayed in Figure S34. After holding at 500 ℃ for 30 minutes, the temperature was increased at 10 ℃ per minute to 800 ℃ and then maintained. It was observed from the figure that during the holding period at 500 ℃, no significant changes occurred in the hydroxyl peak of NC-900. However, throughout the heating process, the hydroxyl peak first decreased and then increased. This phenomenon was attributed to weak desorption of hydroxyl groups during this stage, coupled with an enhancement in peak response intensity as the temperature rose. Furthermore, during the holding at 800 ℃, the peak intensity of the hydroxyl group continuously decreased, indicating that the hydroxyl groups responsible for deactivating the NC-900 catalyst were gradually desorbed at this temperature.

Table S1 The BET pore size characterization and specific surface area data of the C-900, NC-800, NC-900, and NC-100.

**Samples**	**S_{BET}[a] (m^2g^{-1})**	**V_t[b] (cm^3g^{-1})**	**S_{micro}[c] (m^2g^{-1})**	**V_{micro}[d] (cm^3g^{-1})**	**D_a[e] (nm)**
C-900	570.41	0.79	12.5847	0.004	2.79
NC-800	326.06	0.40	44.3035	0.023	2.5
NC-900	402.56	0.41	119.6444	0.057	2.07
NC-1000	326.23	0.46	82.2292	0.042	2.84

[a]S_{BET}: The specific surface area measured by the BET method. V_t[b]: Total pore volume. S_{micro}[c]: Microporous specific surface area. V_{micro}[d]: The micropore pore volume calculated by the t-plot method. D_a[e]: mean pore size mean pore size.

Table S2 The elemental analysis instrument was used to analyze the contents of C, H and N elements in the C-900, NC-800, NC-900 and NC-100 catalysts (wt %).

Sample	C	H	N
C-900	95.13%	0.997%	0%
NC-800	75.92%	2.431%	13.24%
NC-900	80.15%	1.566%	10.66%
NC-1000	86.94%	1.530%	6.07%

Table S3 Toxicity classification based on the Global System for Classification and Labelling of Chemicals.

Toxicity range (mg/L)	Class
LC50/EC50/ChV≤1	Highly toxic
1 < LC50/EC50/ChV≤10	Toxic
10 < LC50/EC50/ChV≤100	Harmful
LC50/EC50/ChV > 100	Harmless

Table S4 The content of Lewis basic sites in the CO_2-TPD curves of NC-900 and C-900 catalysts

entry	NC-900	C-900
wake Basic site	46.87	53.65
medium Basic site	277.31	20.28
strong Basic site	384.15	43.32

To quantitatively describe the Lewis basic sites, CO_2-TPD characterization were performed on the NC-900 and C-900 catalysts, and peak deconvolution was conducted on the TPD curves of both catalysts. The fitting results as shown in Figure S32, were used to quantify the Lewis basic site content of NC-900 and C-900, with the basic sites classified into weak, medium, and strong basic sites according to increasing temperature. It was observed from the figure that the medium and strong basic sites of the NC-900 catalyst exhibited a significant increase. The quantitative results presented in Table S4, indicated that the content of weak basic sites remained almost unchanged, while the medium basic sites increased from 20.28 to 277.31 and the strong basic sites from 43.32 to 384.15, which fully demonstrated that a large number of Lewis basic sites were introduced in the nitrogen-doped NC-900 catalyst.

Table S5 The content of C-OH in XPS and the peak area of hydroxyl group in FTIR of fresh NC-900, used NC-900, annealing NC-900, and $NaBH_4$ treatment of NC-900.

entry	C-OH (%)	OH peak area
Fresh	24.53	60.47
Used	35.2	152.38
Annealing	25.16	72.56
$NaBH_4$	26.31	84.66

The content of C-OH in XPS and the peak area of hydroxyl groups in FTIR were presented in Table S5. After four cycles, the C-OH content in the NC-900 catalyst increased from 24.53% to 35.2%, while the corresponding FTIR hydroxyl peak area rose from 60.47 to 152.38. Following regeneration, both parameters were effectively restored toward their initial levels: the C-OH content decreased to 25.16% and 26.31%, and the peak area dropped to 72.56 and 84.66, after annealing and $NaBH_4$ reduction, respectively. Quantitative analysis of XPS and FTIR indicated that the hydroxyl attachment amount significantly increased after cycling and reverted to near initial levels after the two regeneration methods. Figure S22 and S23 revealed an inverse correlation between surface hydroxyl density and the MB degradation rate in the NC-900/PMS system, indicating that excessive hydroxyl groups on the catalyst surface markedly suppressed the overall catalytic activity.
